# Refining the biomanufacturing of microalgae-derived extracellular vesicles as a potential nanotherapeutic for osteoarthritis

**DOI:** 10.1093/rb/rbag074

**Published:** 2026-04-16

**Authors:** Meng Wang, Sara Gil Izquierdo, Aylin Kara Özenler, Jaqueline Lourdes Rios, Mylène de Ruijter, Debby Gawlitta, Jos Malda, Kenny Man

**Affiliations:** Department of Orthopedics, University Medical Center Utrecht, 3508 GA, Utrecht, The Netherlands; Regenerative Medicine Center Utrecht, University Medical Center Utrecht, 3584 CT, Utrecht, The Netherlands; Regenerative Medicine Center Utrecht, University Medical Center Utrecht, 3584 CT, Utrecht, The Netherlands; Department of Oral and Maxillofacial Surgery & Special Dental Care, University Medical Center Utrecht, 3508 GA, Utrecht, The Netherlands; Department of Orthopedics, University Medical Center Utrecht, 3508 GA, Utrecht, The Netherlands; Regenerative Medicine Center Utrecht, University Medical Center Utrecht, 3584 CT, Utrecht, The Netherlands; Department of Orthopedics, University Medical Center Utrecht, 3508 GA, Utrecht, The Netherlands; Regenerative Medicine Center Utrecht, University Medical Center Utrecht, 3584 CT, Utrecht, The Netherlands; Department of Orthopedics, University Medical Center Utrecht, 3508 GA, Utrecht, The Netherlands; Regenerative Medicine Center Utrecht, University Medical Center Utrecht, 3584 CT, Utrecht, The Netherlands; Department of Clinical Sciences, Faculty of Veterinary Medicine, Utrecht University, 3584 CL, Utrecht, The Netherlands; Regenerative Medicine Center Utrecht, University Medical Center Utrecht, 3584 CT, Utrecht, The Netherlands; Department of Oral and Maxillofacial Surgery & Special Dental Care, University Medical Center Utrecht, 3508 GA, Utrecht, The Netherlands; Department of Orthopedics, University Medical Center Utrecht, 3508 GA, Utrecht, The Netherlands; Regenerative Medicine Center Utrecht, University Medical Center Utrecht, 3584 CT, Utrecht, The Netherlands; Department of Clinical Sciences, Faculty of Veterinary Medicine, Utrecht University, 3584 CL, Utrecht, The Netherlands; Regenerative Medicine Center Utrecht, University Medical Center Utrecht, 3584 CT, Utrecht, The Netherlands; Department of Oral and Maxillofacial Surgery & Special Dental Care, University Medical Center Utrecht, 3508 GA, Utrecht, The Netherlands

**Keywords:** microalgae, extracellular vesicles, nanomedicine, osteoarthritis, sustainable

## Abstract

Osteoarthritis (OA) is a degenerative joint disease characterized by oxidative stress, chronic inflammation and progressive cartilage degradation. Current treatments remain largely symptomatic and fail to target underlying disease mechanisms. Extracellular vesicles (EVs) have emerged as promising nanotherapeutics; however, mammalian-derived EVs face limitations related to cost, scalability and manufacturing complexity. Microalgae represent a sustainable alternative, yet their potential as EV biofactories for regenerative medicine remains largely unexplored. This study investigates the biomanufacturing and therapeutic potential of microalgae-derived EVs for OA. Four microalgae species (*Chlorella sorokiniana*, *Synechococcus sp.*, *Leptolyngbya sp.* and *Chlamydomonas reinhardtii CC1690*) were cultured under varying photoperiods (0, 16 and 24 h light/day) to evaluate effects on viability, growth and EV production. EVs were characterized using transmission electron microscopy, nanoparticle tracking analysis, protein quantification and immunoblotting. Antioxidant activity and therapeutic efficacy were assessed in a cytokine-induced OA-like *in vitro* model. All species maintained high viability (>80%), with EV yield strongly dependent on light exposure. *Leptolyngbya sp*. demonstrated the fastest growth and highest EV production under extended illumination, generating EVs with antioxidant properties. *Leptolyngbya*-derived EVs enhanced mesenchymal stromal cell proliferation and migration and mitigated cytokine-induced matrix degradation. These findings establish *Leptolyngbya* as a scalable, cost-effective source of therapeutic EVs for OA.

## Graphical Abstract

**Graphical Abstract:**
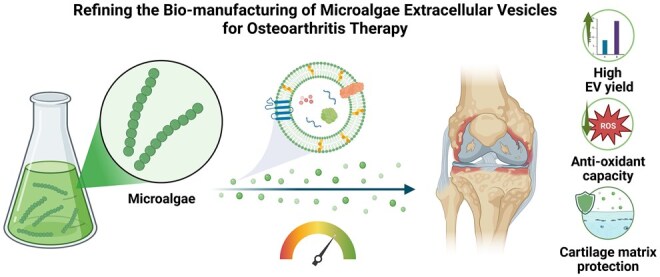

## Introduction

Osteoarthritis (OA) is a chronic degenerative joint disease, affecting millions and causing significant disability. Characterized by pain, dysfunction and joint deformity, OA places a significant burden on healthcare systems and severely diminishes patients’ quality of life [1, 2]. The global prevalence of OA was estimated at 7.96% of the global population in 2020 [3], which is projected to rise due to our growing, aging population, thus incurring substantial economic costs related to treatment, rehabilitation and lost productivity [4, 5]. OA is a multifactorial disorder shaped by a complex interplay of intrinsic and extrinsic factors, including age, sex, genetic predisposition, obesity, prior joint injury, diet and lifestyle [6, 7]. There is currently no standard-of-care for OA, where disease management relies on conservative approaches such as corticosteroid injections, non-steroidal anti-inflammatory drugs, physical therapy or lifestyle changes [8]. Surgical treatments such as microfracture and mosaicplasty can alleviate pain [9], however often result in unsatisfactory long-term outcomes [1, 10]. Currently, there is no cure for OA, highlighting the urgent need for novel therapies that address the underlying cause of the disease. While the precise etiology of OA remains elusive, accumulating evidence points to a confluence of factors, including chronic inflammatory signaling cascades [11], oxidative stress [12], chondrocyte apoptosis [13] and dysregulated energy metabolism [14]. Thus, the development of novel therapeutic interventions targeting the modulation of these factors represents a promising avenue for the development of disease-modifying OA treatments.

Cell-based strategies utilizing mesenchymal stem/stromal cells (MSCs) have shown promise due to their immunomodulatory capabilities [15, 16]. Although promising, their clinical translation is hampered by significant hurdles, including poor cell survival, immune rejection, ethical issues, complex regulatory approval, high production cost and potential tumor formation [17, 18]. Nanotechnology offers a powerful solution to these challenges, enabling targeted delivery, improved biocompatibility, the ability to traverse biological barriers (e.g. blood-brain barrier), off-the-shelf storage and minimized adverse immune responses [19–21]. Extracellular vesicles (EVs) are emerging as a promising nanoscale therapy for musculoskeletal repair, overcoming limitations associated with cell-based therapies. EVs are cell-secreted lipid nanoparticles, which contain a package of bioactive molecules (i.e. proteins, metabolites, nucleic acids) and play a crucial role in intercellular communication [20, 22–24]. By eliminating the risks of uncontrolled differentiation, tumorigenicity and poor survival associated with cell therapies, EV-based therapies offer an exciting prospect for a readily available, off-the-shelf OA treatment [25–27]. Moreover, EVs can readily encapsulate and deliver various therapeutic molecules (e.g. anti-inflammatory drugs, growth factors) directly to the defect site, enhancing their efficacy and reducing side effects [28, 29]. Moreover, the economic advantage of EV-based approaches in comparison to MSC therapies has been reported, with a dose of MSC-EVs (10^11^ particles) costing up to €3082, whilst clinical doses of MSCs can cost up to €42 673 depending on dose size and production scale [30].

Despite their therapeutic and economic appeal, the clinical translation of mammalian cell-derived EVs remains limited by several practical challenges, including donor variability, low production yields, labor-intensive isolation procedures and difficulties in large-scale manufacturing and long-term storage [18, 31, 32]. In contrast, microalgae constitute a sustainable and renewable source of bioactive compounds widely utilized in the health, cosmetics and food industries [33, 34]. Microalgae are photosynthetic autotrophic microorganisms, rich in bioactive metabolites, such as pigments, vitamins and antioxidants [33, 35]. Critically, algae cultivation is scalable, cost-effective and environmentally friendly, overcoming the limitations of mammalian cell-based production [36, 37]. Therefore, microalgae could provide a robust renewable platform for generating therapeutic EVs at clinically relevant scales. Despite emerging interest in the biomedical applications of microalgae and their derivatives, their potential as a platform for producing therapeutic EVs remains underexplored. In particular, the refinement of algal cultivation strategies to optimize EV yield and quality has received little attention. Given that microalgae are highly sensitive to environmental conditions, factors such as light intensity, nutrient availability and temperature can significantly influence vesicle production [38, 39], critical variables that must be controlled to meet clinical-grade standards.

**Figure 1:**
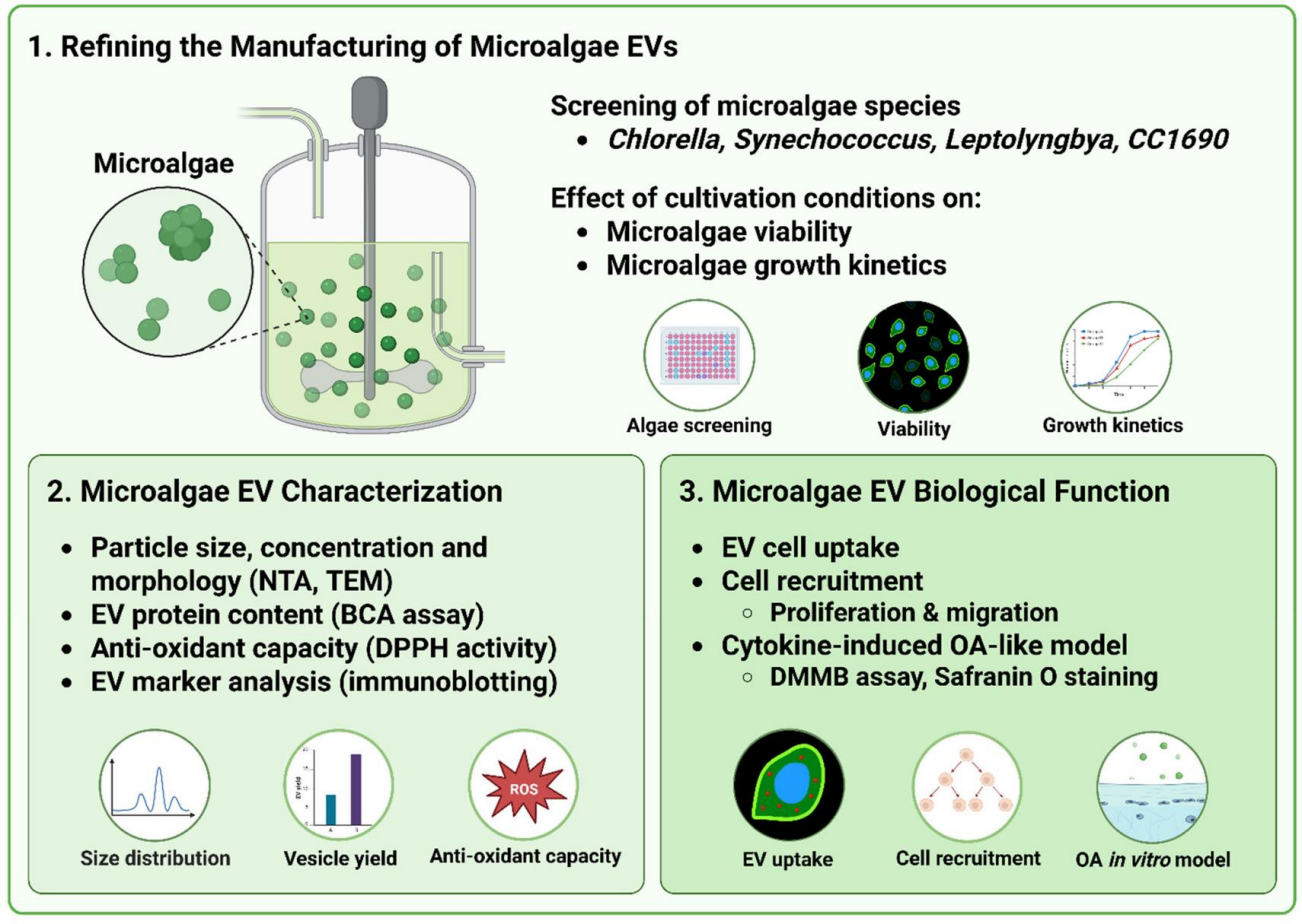
Experimental overview investigating the effect of biomanufacturing conditions on microalgae EV production and therapeutic potency. (**1**) The influence of different manufacturing conditions (i.e. light exposure, harvesting frequency) on microalgae viability, growth kinetics and yield. *Chlorella sorokiniana* (*Chlorella*)*, Synechococcus* sp. (*Synechococcus*), *Leptolyngbya* sp. (*Leptolyngbya*)*, Chlamydomonas reinhardtii CC1690* (*CC1690*). (**2**) EVs were isolated from microalgae over a 2-week period, and the nanoparticles were characterized by their size distribution, morphology, protein content, antioxidant capacity and EV marker expression. (**3**) Investigating the influence of microalgae EV treatment on recipient cell uptake, proliferation, migration and anti-catabolic properties in an OA-like *in vitro* model.

This study aims to optimize the biomanufacturing of microalgal EVs and to evaluate these as a potential next-generation nanotherapeutics for OA. The research specifically focuses on selecting a potent microalgal species with a high EV production yield, evaluating the effects of different light regimes on algal growth and EV biogenesis and determining how medium harvest frequency influences EV yield and quality (Figure 1). The most productive and scalable species were subsequently chosen for further investigation, where the potency of its EVs was assessed for their ability to mitigate inflammation-induced cartilage-like ECM degradation in a cytokine-induced OA-like *in vitro* model.

## Materials and methods

### Microalgae cultivation

*Chlorella sorokiniana*, *Synechococcus* sp., *Leptolyngbya* sp. and *Chlamydomonas reinhardtii CC1690* with initial concentration of 25 × 10^6^ cells/mL were cultured separately in modified M8 medium, sterile artificial seawater medium and modified Tris-Acetate-Phosphate (TAP) medium (Supplementary Table S1) on shakers (Multitron, Infors HT, Switzerland) with 150 rpm of agitation speed. All the microalgal cultivation media were supplemented with HEPES (20 mM) to stabilize pH. Bright white LED (LedstripKoning, HWCS600-03M) was used as artificial light source, and different photoperiods (0, 16 and 24 h of light/day) were applied accordingly to stimulate microalgae growth. The light intensity was approximately 100 μmol photons m^−2^ s ^−1^. All the microalgal cultures were maintained for 2 weeks, with medium changes occurring once or twice a week, unless otherwise stated. Briefly, microalgal cultures were harvested and centrifuged at 1200*g* for 5 min, the resulting microalgae pellets were resuspended in fresh media and returned to the original flasks/shakers for further cultivation.

### Microalgae growth kinetics and viability

To determine microalgal number and biomass, the optical density at 750 nm (OD_750_) was measured (Supplementary Tables S2 and S3). For each measurement, a total of 100 µL of diluted sample was placed into a 96-well microplate and read using a microplate reader (CLARIOstar Plus, BMG LABTECH, Ortenberg, Germany). To assess the growth kinetics of each microalgal species, normalized microalgal number (Nt) and biomass (Bt) were calculated via Equations (1) and (2)


(1) $$N_{t}=\frac{N_{\mathrm{day}\;14}}{N_{\mathrm{day}\;0}}$$

(2) $$B_{t}=\frac{B_{\mathrm{day}\;14}}{B_{\mathrm{day}\;0}}$$

where *N_t_* represents the normalized microalgal cell number, and *N*_day 14_ and *N*_day 0_ are the microalgal cell number after 2 weeks and the initial culture microalgal cell number, respectively. *B_t_* represents the normalized microalgal dry biomass, and *B*_day 14_ and *B*_day 0_ are the microalgal biomass after 2 weeks and the biomass of initial microalgal used for cultivation, respectively.

The viable microorganisms can be traced by the autofluorescence of chlorophyll under red wavelength, which was detected with an excitation/emission of 640/700 nm. The non-viable microalgae were stained by SYTOX™ Orange (1 µM, Invitrogen™) and detected with an excitation/emission of 543/570 nm. At least six images of each cultivation condition were taken by Thunder microscopy (Leica Microsystems, Germany). Total area of both live and dead microalgae signals was quantified via Fiji ImageJ (https://imagej.net/software/fiji/) processing. Viability of microalgae was calculated as a percentage via Equation (3), where TA_l_ and TA_d_ represent total area of live and dead microalgae separately


(3) $$\mathrm{Viability}\;(\%)=\frac{TA_{l}}{{\mathrm{TA}}_{l}{+\mathrm{TA}}_{d}}\times 100\%$$

### Microalgae EV isolation and characterization

EVs were initially isolated from microalgae-conditioned medium using sequential centrifugation at 10 000*g* followed by 120 000*g* to evaluate vesicle recovery across fractions. Based on this initial optimization, subsequent experiments utilized the 10 000*g* EV fraction (Supplementary Figure S1). The collected microalgae conditioned medium was centrifuged at 3000*g* for 20 min to remove residual microalgal cellular debris. The supernatant was then filtered through a 0.22 µm membrane filter and stored at −80°C for subsequent EV isolation. The microalgae conditioned media was centrifuged at 10 000*g* for 30 min at 4°C using an Optima XE-90 ultracentrifuge with a SW 32 Ti rotor (Beckman Coulter, USA). The resulting EV pellet was washed in sterile phosphate-buffered saline (PBS) and centrifuged following the same conditions. The EV pellet was then resuspended in PBS and stored at −80°C until required. UV–Vis absorbance spectra of lysed EVs was performed using the NanoDropUltra Spectrophotometer (Thermo Scientific).

To estimate EV particle size and concentration, five videos (30 sec each) were recorded using a NanoSight NS500 instrument (Malvern Panalytical, Great Malvern, UK) equipped with a sCMOS camera and a 405 nm blue laser, at 25°C. Samples were diluted between 1:100 and 1:1000 in PBS to obtain a particle number in the chamber between 20 and 100 to ensure optimal detection and tracking. Data were processed using NTA software version 3.4.

EVs were imaged with a FEI Tecnai 12 transmission electron microscope (Thermo Fisher Scientific, Waltham, MA, USA), operated at 80 kV high tension with image acquisition via the Tecnai User Interface software. The samples were placed in a carbon-coated copper grid and negatively stained. Briefly, grids were incubated in 2% uranyloxalate-acetate (pH 7) for 4 min and then in a solution containing 0.1% (w/v) methyl cellulose and 2% (w/v) uranyl acetate (pH 4) on ice for 5 min. After staining, excess solution was gently removed with filter paper and grids were air-dried before imaging.

Total EV protein concentration was determined using the Micro BCA Protein Assay Kit (Thermo Scientific, 23235) according to the manufacturer’s instructions. Briefly, 150 µL of sample and working reagent was added per well and incubated at 37°C for 2 h. Absorbance was measured at 562 nm on a microplate reader (CLARIOstar Plus, BMG LABTECH, Ortenberg, Germany).

Immunoblotting analysis was conducted to assess the presence of EV-associated protein markers. The Exo-Check™ Antibody Array (System Biosciences, EXORAY200B-4), which detects EV markers (CD63, CD81, ALIX, FLOT1, ICAM1, EpCam, ANXA5 and TSG101), was employed following the manufacturer’s instructions. Briefly, 90 µg of EV protein was incubated within the membrane at 4°C overnight. Following which, the membrane was developed using the SuperSignal™ West Pico PLUS Chemiluminescent Substrate (Thermo Scientific, 34577) and images acquired with the ChemiDoc Imaging System (Bio-Rad Laboratories, Hercules, USA).

2,2-Diphenyl-1-picrylhydrazyl (DPPH) assay was used to determine the total antioxidant potential of EV samples [40, 41]. A total of 75 µL of DPPH• solution (50 µM in methanol) was added to 25 µL of EV samples containing 90 µg/mL of protein. Methanol was used for the baseline correction. A total of 100 µg/mL of ascorbic acid was used as a positive control. The mixture was allowed to stand for 2 mins at room temperature. The change in absorbance was measured at 515 nm using the microplate reader (CLARIOstar Plus, BMG LABTECH, Ortenberg, Germany). The ability of DPPH• radical scavenging activity was calculated by using Equation (4), where *A*0 is the absorbance of the control and *A*1 is the absorbance of the EV sample


(4) $$\mathrm{Scavenging}\;\mathrm{effect}\;(\%)=\frac{A0-A1}{A0}\times 100\%$$

### Mammalian cell cultures

Human bone marrow-derived mesenchymal stromal cells (hBMSCs) were isolated from bone marrow aspirates of patients after informed consent, in accordance with a biobank protocol approved by the local Medical Ethics Committee (TCBio-08-001, University Medical Center Utrecht) [42]. hBMSCs were cultured in basal medium which consisted of minimal essential medium (α-MEM; Gibco, 12571063) supplemented with 10% heat-inactivated fetal bovine serum (FBS) (CAPRICORN, CP24-7059-HI), 0.2 mM L-ascorbic acid-2-phosphate (Sigma-Aldrich, 1713265-25-8), 100 U/mL penicillin with 100 mg/mL streptomycin (Gibco, 15140) and 1 ng/mL basic fibroblast growth factor (bFGF) (R&D Systems, 233-FB-500). The basal medium was replaced twice per week. The cells were passaged when 80% confluency was reached using 0.25% Trypsin-EDTA (Gibco, 25200072). At passage 4, hBMSCs were used for subsequent experiments. Basal medium, supplemented with EV-free FBS (depleted of EVs by ultracentrifugation at 120 000*g* for 16 h), was added to the cells with/without microalgae EVs according to experimental design.

The chondrogenic cell line, ATDC5s, was cultured in basal medium composed of DMEM/F-12 (Gibco, 11320033), supplemented with 5% FBS and 100 U/mL penicillin with 100 µg/mL streptomycin (Gibco, 15140). For monolayer chondrogenic differentiation, 2.1 × 10^5^ cells per well were plated in a 48-well plate (Greiner) and cultured in chondrogenic medium consisting in high-glucose DMEM (Gibco, 31966), 1% ITS Premix (Corning, 354352), 40 µg/mL L-proline (Sigma-Aldrich, P5607), 10 ng/mL TGF-β (PeproTech, 100-21), 0.1 µM dexamethasone (Sigma-Aldrich, D4902), 50 µg/mL ascorbate 2-phosphate (Sigma-Aldrich, A8960) and 100 U/mL penicillin with 100 µg/mL streptomycin (Gibco, 15140). For pellet chondrogenic differentiation, 2 × 10^5^ of ATDC5 per well were plated in a 96-well U-bottom plates (Greiner, E22073N3), centrifuged at 300*g* for 4 min and cultured in ATDC5 chondrogenic medium.

### EV uptake assay

To visualize microalgal EVs uptake by mammalian cells, hBMSCs were treated with 20 µg/mL of CellMask™ (Invitrogen, C10046) labeled *Leptolyngbya-EVs* (Lepto-EVs) as previously described [43]. Briefly, Lepto-EVs were incubated for 10 min in dark conditions in CellMask™ solution (1:1000 in PBS). EVs were centrifuged at 120 000*g* for 70 min to remove the unbound dye using an Optima XE-90 ultracentrifuge with a SW 32 Ti rotor (Beckman Coulter, USA). Labeled-EVs were resuspended in PBS and added to hBMSCs, which were seeded in 48-well plate and cultured overnight in expansion medium (containing EV-free FBS), and incubated for 16 h at 37°C and 5% CO_2_. hBMSCs were washed twice with PBS, then fixed with 10% formalin for 10 min at room temperature. After two washes with PBS, cells were permeabilized with 0.1% Triton X-100 for 10 min. After washing twice with PBS, samples were blocked with 2% BSA in PBS for 2 min. Blocking solution was removed and samples were incubated with 2.5 µg/mL of fluorescently conjugated phalloidin (in 2% BSA) for 20 min in the dark. After washing twice with PBS, nuclei were stained with 10 ng/mL of DAPI (in PBS) for 5 min in the dark, washed with PBS and images were acquired using confocal microscopy (Leica Microsystems, Germany). The excitation/emission wavelengths of DAPI, phalloidin and CellMask™ were 410/494, 506/634 and 669/754 nm, respectively.

### hBMSCs proliferation and migration

The effects of Lepto-EVs on hBMSCs proliferation were assessed via quantification of metabolic activity and DNA content. Briefly, hBMSCs (1 × 10^4^ cells/cm^2^) were treated with basal medium supplemented with or without 2 µg/mL of Lepto-EVs and incubated for 1, 3 and 7 days. Cells cultured in basal medium was used as the control. At each time point, metabolic activity and DNA content were evaluated. To assess metabolic activity, the AlamarBlue reagent (44 mM of resazurin sodium salt, Sigma-Aldrich, R7017) was added and incubated for 4 h at 37°C in the dark. Fluorescence was measured in a microplate reader (CLARIOstar Plus, BMG LABTECH, Ortenberg, Germany) using an excitation/emission of 530/590 nm. To assess DNA content, the Quant iT™ PicoGreen^®^ dsDNA kit (Thermo Fisher Scientific, P11495) was used according to the manufacturer’s instructions. Briefly, cells were washed twice with PBS, then lysed with 0.1% Triton-X solution in PBS, followed by three cycles of freeze–thaw. Samples were diluted in TE buffer (10 mM Tris-HCl, 1 mM EDTA, pH 7.5) and transferred to a 96-well plate (100 µL per well), after which 100 µL of the PicoGreen working reagent was added and plates were incubated for 5 min at room temperature in the dark. Fluorescence was measured using the mentioned microplate reader with an excitation/emission of 485/520 nm.

To assess migration rate, the scratch assay was performed as previously described [44]. Briefly, cells were cultured in 6-well plates (30 × 10^3^ cells/cm^2^) in basal medium for 24 h. A linear scratch was made using a sterile 20 µL pipette tip. After a PBS wash, basal medium (containing EV-depleted FBS) supplemented with/without EVs were added to the cells. Images were captured at day 0, 1 and 2 using an inverted microscope (DMi1, Leica Microsystems, Wetzlar, Germany). The scratch area was quantified using the software FIJI ImageJ, the percentage of closure was calculated with the following formula [5], where *A*0 is the area immediately after creating the scratch and *At* is the area at day 1


(5) $$\mathrm{Closure}\;(\%)=\;\frac{A0-At}{A0}\;x\;100\%$$

### Assessment of antioxidant activity of Lepto-EVs in OA-like model

Intracellular ROS levels were measured using 2,7-dichlorofluorescein diacetate (DCFDA, Sigma-Aldrich, 287810). This non-fluorescent probe is converted to a fluorescent product in the presence of ROS, with fluorescence intensity directly proportional to ROS levels, enabling quantitative assessment of oxidative stress. ATDC5 cells were plated in a 96-well plate at a density of 5 × 10^3^ cells/well and incubated at 37°C with 5% CO_2_ in DMEM medium without phenol red (Gibco, 11054020). After 24 h, cells were treated with Lepto-EVs (2 µg/mL) and 10 ng/mL IL-1β (PeproTech, 200-01B) or 10 ng/mL TNF-α (PeproTech, 300-01A). As a negative control, untreated cells were treated with fresh medium. After 24 h, cells were incubated with DCFDA, at a concentration of 40 µM diluted in medium without FBS for 45 min at 37°C with 5% CO_2_. Fluorescence was measured using the previously mentioned microplate reader with an excitation/emission of 485/535 nm.

### Cytokine-induced OA-like model

To evaluate the anti-catabolic effects of EV treatment, a cytokine-induced OA-like inflammatory model using the chondrogenic cell line ATDC5s was established. ATDC5s cultured in chondrogenic medium, as monolayers or as pellets, were exposed to either 10 ng/mL IL-1β (PeproTech, 200-01B) or 10 ng/mL TNF-α (PeproTech, 300-01A) [45]. For the EV treated groups, ATDC5s were treated with 2 µg/mL of Lepto-EVs. Cytokines and/or EVs were added fresh at each medium change.

Safranin-O staining was conducted to visualize cartilage-like glycosaminoglycan (GAG) formation. For chondrogenically differentiated ATDC5 monolayers, samples were stained with a modified Safranin-O protocol [46]. Briefly, ATDC5 monolayers were washed twice with PBS and fixed with 10% formalin for 20 min. After three washes with PBS, samples were incubated with 0.1% Safranin-O solution (Sigma Aldrich, 33209) for 30 min. Following three washes with PBS, the samples were imaged using a stereoscopic microscope (Olympus SZ61, Olympus Corporation, Tokyo, Japan).

For ATDC5 chondrogenic pellets, samples were washed with PBS and placed in a drop of 5% low melt agarose (SeaKem^®^LE Agarose, Lonza), fixed in 10% formalin overnight and dehydrated by sequential immersion in: 70% ethanol (1 h), 90% ethanol (1 h), 96% ethanol (twice, 1 h each), 100% ethanol (1 h), 100% ethanol overnight and Xylene (twice, 1 h each). Samples were then embedded in paraffin and sectioned in 5 µm using a rotatory microtome (HM 340E, Thermo Fisher Scientific, Waltham, MA, USA). Sections were deparaffinized and hydrated by sequential immersion in Xylene (twice, 5 min each), 100% ethanol (twice, 3 min each), 96% ethanol (twice, 3 min each), 70% ethanol (twice, 3 min each) and distilled water (three times for 2 min each). Safranin O/Fast Green staining was performed by placing the sections in Weigert’s hematoxylin for 5 min, washed under running tap water for 10 min and rinsed in distilled water. Sections were then counterstained with 0.4% aqueous Fast Green (MP Biomedicals, 0219517825) for 4 min. The slides were immersed in 0.125% Safranin O solution (Sigma-Aldrich, 33209) for 5 min after rinsing twice with 1% acetic acid (1 and 4 min, respectively). Subsequently, slides were quickly rinsed three times with 100% ETOH changes and transferred into Xylene for 5 min before mounting. Images were taken using a bright-field optical microscope (Olympus BX51, Olympus Corporation, Tokyo, Japan).

Dimethyl methylene blue (DMMB) assay was used to quantify GAG production. For monolayer samples, 100 µL of Milli-Q water was added to each well, then three freeze–thaw cycles were applied, and samples were subsequently incubated overnight at 60°C in 100 µL of papain solution (papain 250 µg/mL; cysteine HCl 1.57 mg/mL) prepared in 2× papain buffer (0.2 M sodium phosphate, 10 mM EDTA, pH 6.0). To quantify GAGs, papain-digested samples were diluted 1:30 in PBS-EDTA (40 mM Na_2_HPO_4_, 53.1 mM NaH_2_PO_4_·2H_2_O, 10 mM disodium EDTA), transferred in duplicate of 50 µL per well to a 96-well plate and combined with 100 µL of DMMB solution (50 µM 1,9 dimethyl methylene blue, 40.6 mM NaCl, 40.5 mM glycine, 0.5% v/v ethanol, pH 3). The absorbance was measured at 525 and 595 nm wavelengths with previously mentioned microplate reader. For ATDC5 pellets, samples were first freeze-dried and then digested with the previously mentioned papain solution.

### Statistical analysis

All statistical analyses were performed using GraphPad Prism 10 (GraphPad Software, San Diego, CA, USA). When comparing two groups, data were analysed using Student’s *t*-test after confirming for equality of variances with an *F*-test. For experiments involving more than two groups, one-way ANOVA was performed followed by multiple comparisons. In the case which involves two independent variables, two-way ANOVA were performed. All error bars are mean ± SD of at least three independent experiments. Statistically significant *P*-values are indicated in figures and legends as *P ≤ 0.05, **P ≤ 0.01, ***P ≤ 0.001 and ****P ≤ 0.0001, ns represents no significant difference (*P* > 0.05).

## Results

### The effects of light exposure conditions on microalgae cultivation

**Figure 2:**
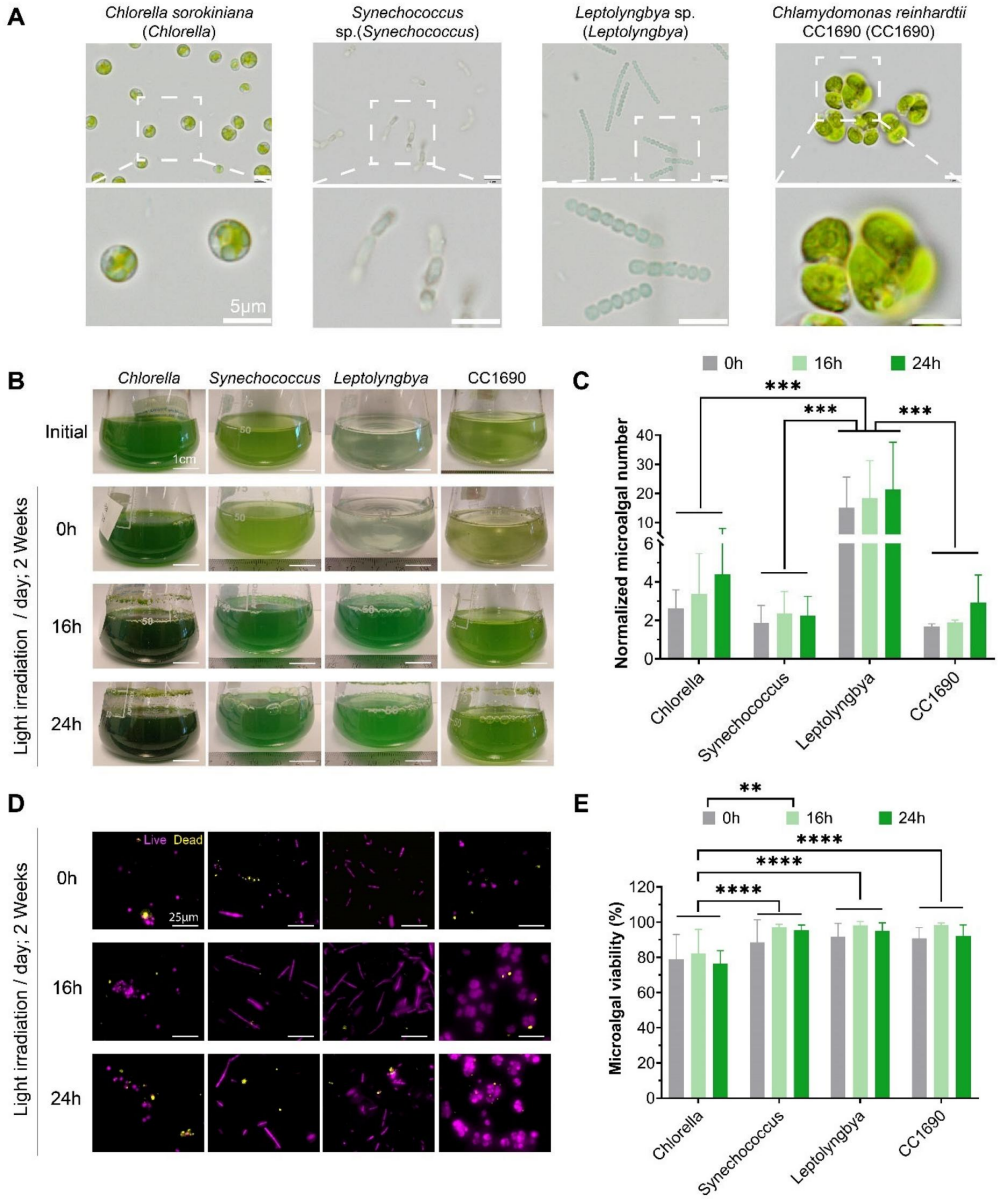
The effect of photoperiod on the growth and survival of different microalgae species. (**A**) Microscopic images of different microalgae species. The effects of different cultivation conditions by 2 weeks, on (**B**) microalgae medium pigmentation, (**C**) normalized microalgae number, *N_t_* (*n* = 3), (**D**) live/dead fluorescent imaging of microalgae, (**E**) microalgae viability (*n* = 6). Data expressed as mean ± SD. **P ≤ 0.01, ***P ≤ 0.001 and ****P ≤ 0.0001.

To refine the manufacture of microalgae EVs, we initially determined the influence of the cultivation conditions on the viability and growth kinetics of different microalgae species. The cultivation conditions involved a photoperiod of either 0, 16 or 24 h per day, for a total period of 2 weeks. Figure 2A shows the microscopic images of the different microalgae species assessed, showcasing their distinct morphological features. Macroscopic images highlight the impact of increased exposure of light irradiation on the intensity of pigmentation within the medium of different microalgae cultures following 2 weeks cultivation (Figure 2B). A light exposure-dependent increase in microalgae number was observed in all species assessed, with *Leptolyngbya* exhibiting significantly increased microalgae number compared to *Chlorella* (4.8-fold), *CC1690* (7.3-fold) and *Synechococcus* (9-fold) (*P* ≤ 0.001) (Figure 2C). A similar normalized microalgae biomass profile was also observed (Supplementary Figure S2). The impact of light irradiation on microalgae viability was assessed through live/dead staining and semi-quantification (Figure 2D and E). Our results show that all microalgae species exhibited high viability (≥80%) after 2 weeks culture in different light irradiation periods, with *Chlorella* exhibiting significantly lower viability when compared to *Synechococcus*, *Leptolyngbya* and *CC1690* (*P* ≤ 0.0001). The 16 h light irradiation exhibited the highest microalgae viability when compared to the 0 h (*P* ≤ 0.01) and 24 h light cycles (*P* > 0.05) after 2 weeks culture.

### The influence of light exposure conditions on microalgae EV production

**Figure 3:**
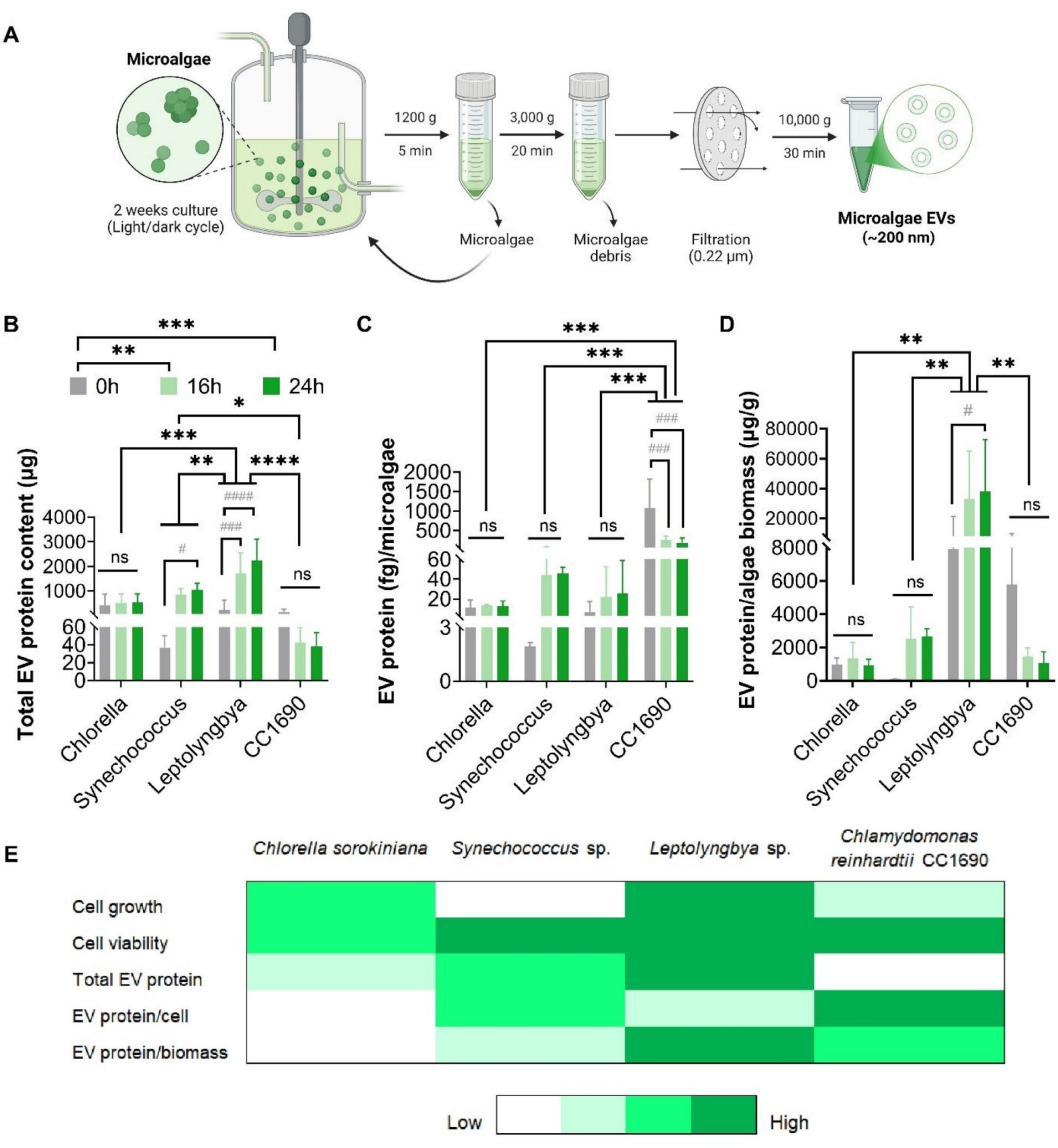
The effect of microalgae biomanufacturing conditions of EV production. (**A**) Schematic overview of the microalgae cultivation and EV isolation procedure. Quantification of EV production by (**B**) total EV protein content, (**C**) EV protein per microalgae, (**D**) EV protein per microalgae biomass. (**E**) Summary of the key outputs regarding microalgae cultivation and EV production. Data expressed as mean ± SD. *P ≤ 0.05, **P ≤ 0.01, ***P ≤ 0.001 and ****P ≤ 0.0001. ^#^*P* ≤ 0.05, ^###^*P* ≤ 0.001 and ^####^*P* ≤ 0.0001 indicate significant differences among light conditions within the same microalgae species. ns = not significant, *P* > 0.05.

We next evaluated the influence of microalgae light exposure conditions on EV production yield. Figure 3A provides an overview of the microalgae cultivation and EV isolation/purification used in this study. Our findings showed that all microalgae species were able to secrete EVs during the different cultivation conditions (Figure 3B). *Leptolyngbya* exhibited the highest EV protein content, which was significantly higher when compared to *Synechococcus* (∼2.16-fold, *P* ≤ 0.01), *Chlorella* (∼4.14-fold, *P* ≤ 0.001) and *CC1690* (∼15.27-fold, *P* ≤ 0.001). A light-dependent increase in EV protein content was observed for *Synechococcus* and *Leptolyngbya*, whilst light exposure negatively affected the production of EV protein per microalgae from *CC1690*. For *Chlorella*, light exposure did not affect EV protein content (Figure 3B). In more detail, for *Synechococcus*, 24 h light irradiation significantly increased EV protein content compared to 0 h light exposure (*P* ≤ 0.05); for *Leptolyngbya*, both 16 h (*P* ≤ 0.001) and 24 h (*P* ≤ 0.001) exhibited significantly increased EV protein yields when compared to 0 h light exposure; for *CC1690*, no effect on EV protein yields was observed while increasing light irradiation (*P* > 0.05).

To determine the influence of cultivation conditions on the EV production per microalgae, EV protein content was normalized with the microalgae number (Figure 3C) and microalgae biomass (Figure 3D, biomass shown in Supplementary Figure S1). Following normalization with microalgae number, *CC1690* exhibited the highest EV protein content, which was significantly higher than the *Synechococcus* (*P* ≤ 0.001), *Leptolyngbya* (*P* ≤ 0.001) and *Chlorella* (*P* ≤ 0.001) groups (Figure 3C). Normalization with microalgae biomass, *Leptolyngbya* displayed the highest EV protein yield, followed by *CC1690* (*P* ≤ 0.01), *Synechococcus* (*P* ≤ 0.01) and *Chlorella* (*P* ≤ 0.01) (Figure 3D).The influence of different light irradiation cycles on the EV production yield within each microalgae species remains consistent to the results shown in Figure 3B. Figure 3E provides an overview of important outputs influencing the selection of an optimum microalgae EV factory, such as microalgae growth kinetics, viability and EV production yields. *Leptolyngbya* exhibited high algae growth kinetics, whilst *Leptolyngbya*, *Synechococcus* and *CC1690* displayed high cell viability. Total EV production yield was highest in the *Leptolyngbya* group, where normalization with cell number and biomass, CC1690 and *Leptolyngbya*/*CC1690* exhibited the highest EV protein values, respectively. Taken together, *Leptolyngbya-*EVs were selected for further analysis due to the increased growth kinetics, viability and EV production yields.

### Characterization of *Leptolyngbya-*derived EVs

**Figure 4:**
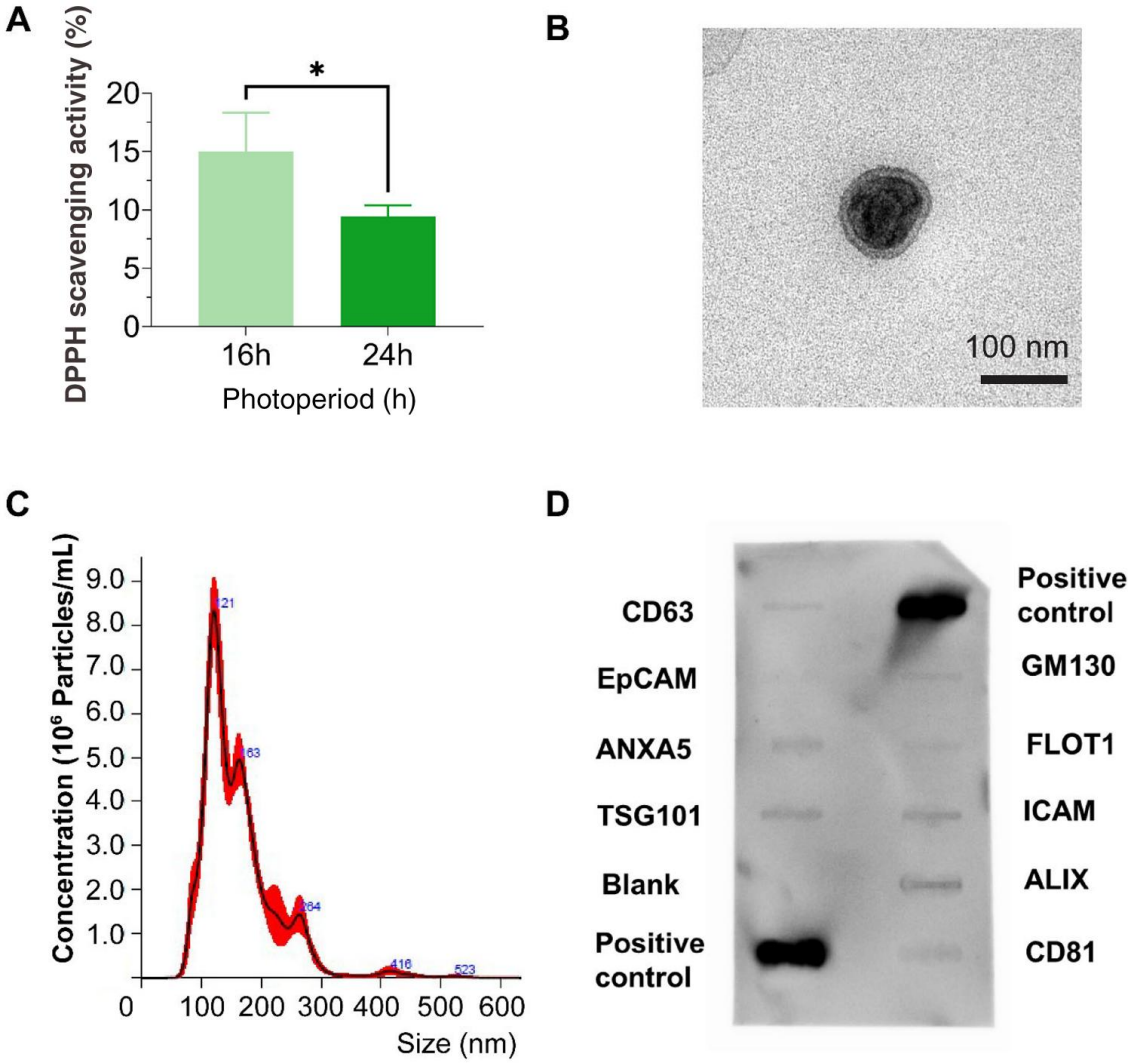
Characterization of *Leptolyngbya*-derived EVs. (**A**) Percentage of DPPH scavenging capacity of EVs cultured in different light/dark cycles (*n* = 3) (**B**) TEM, (**C**) NTA and (**D**) quality assessment of isolated EVs with the Exo-Check™ antibody array. Lepto-EVs were assayed to detect eight EV-associated protein markers, including CD63, ANXA5 (annexin A5), CD81, FLOT1 (flotilin-1), ICAM1 (intercellular adhesion molecule 1), ALIX (programmed cell death 6 interacting protein), TSG101 (tumor susceptibility gene 101), EpCAM (epithelial cell adhesion molecule) and a control for cellular contamination, GM130 (cis-golgi matrix protein). Data expressed as mean ± SD. *P ≤ 0.05.

Following the selection of *Leptolyngbya* as our microalgae EV source, we further explored refining our manufacturing conditions to improve EV production yields. Due to the importance of light exposure to *Leptolyngbya* growth kinetics, we initially assessed the influence of light irradiation cycles (16 and 24 h) on the antioxidant capacity of isolated EVs through the DPPH• scavenging assay. Our findings showed that EVs produced from the 16 h light irradiation cycle exhibited a 1.59-fold increase in the DPPH• scavenging activity (15%) when compared to the 24 h group (9.44%) (*P* ≤ 0.05) (Figure 4A). Moreover, we confirmed that the EV from the different microalgae EV sources exhibited a similar degree of DPPH scavenging capacity (*P* > 0.05) (Supplementary Figure S3). To assess the potential cargo that likely contributed to the antioxidant value of Lepto-EVs, we performed UV–Vis absorption spectroscopy on lysed Lepto-EVs. In our analysis, the spectra revealed a noticeable peak at about 210 nm (Supplementary Figure S4), likely indicating phenolic/flavonoid substances, which possess strong free-radical scavenging and anti-inflammatory properties.

The influence of microalgae medium collection frequency on the EV production yield was evaluated. The quantity of EVs produced from the ‘Standard’ cultivation protocol (one collection after 2-week cultivation) and a ‘Frequent’ cultivation protocol (four collections during 2-week cultivation) was assessed. Our findings showed that when compared to the ‘Standard’ cultivation protocol used for microalgae cultivation, our ‘Frequent’ harvesting regimen substantially increased vesicles production yield regarding EV protein content and particle number by 2.64-fold and 270-fold, respectively (Supplementary Figure S4). The vesicles produced from the ‘Frequent’ harvesting regimen were slightly larger (161.7 ± 62.2 nm) than those obtained from the ‘Standard’ cultivation protocol (104.3 ± 58.5 nm), although there was substantial overlap in size distributions (Supplementary Figure S5). Moreover, TEM imaging showed vesicles in both the groups exhibited similar morphology and size distributions (Supplementary Figure S5). The 16 h light irradiation cycle in conjunction with the ‘Frequent’ harvesting regimen was employed for the rest of this study. Following the isolation of EVs, TEM imaging showed the isolated nanoparticles exhibited a typical spherical morphology with a heterogeneous size ranging from ∼50 to 300 nm (Figure 4B). NTA analysis showed that the isolated Lepto-EVs exhibited an average diameter of 161.7 ± 62.2 nm and a total concentration of 1.21 × 10^11^ particles/mL (Figure 4C). To confirm the isolated nanoparticles were EVs, immunoblotting was conducted using the Exo-Check™ Exosomes Antibody Array. Immunoblotting confirmed the presence of EV-associated markers (CD63, EpCAM, ANXA5, TSG101, FLOT1, ICAM, ALIX and CD81) (Figure 4D).

### Lepto-EVs promote the proliferation and migration of hBMSCs

**Figure 5:**
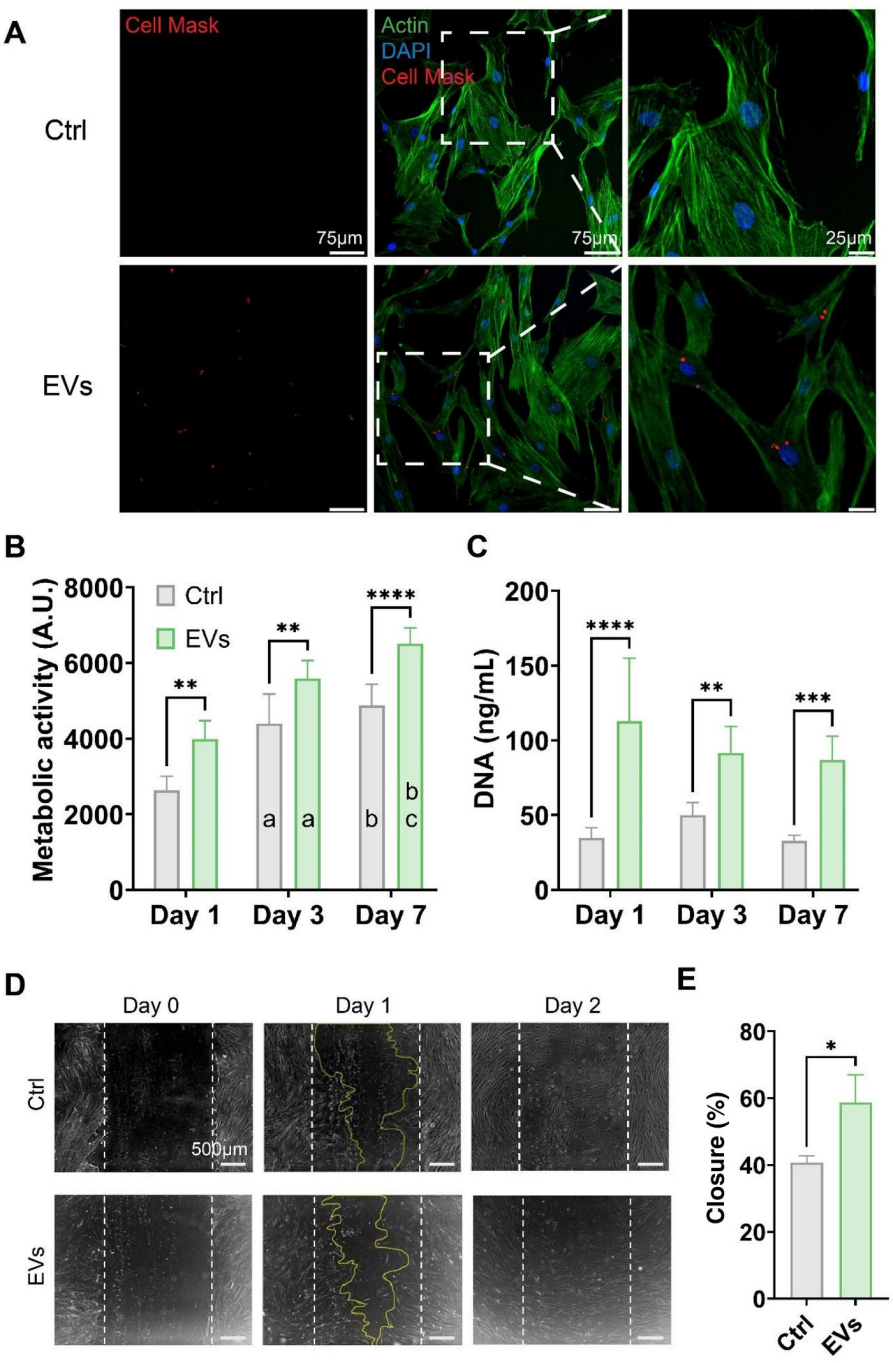
The influence of Lepto-EVs on hBMSCs general behavior. (**A**) Fluorescent images of Cell Mask-labeled Lepto-EV uptake by hBMSCs. The effects of Lepto-EVs on hBMSCs (**B**) metabolic activity, (**C**) DNA content and (**D**) migration microscopic images, (**E**) quantified % gap closure at 1 day. Data expressed as mean ± SD. *P ≤ 0.05, **P ≤ 0.01, ***P ≤ 0.001 and ****P ≤ 0.0001. Letters (**a**–**c**) indicate significant differences within the same group (at least *P ≤ 0.05) between (**a**) day 1 and day 3, (**b**) day 1 and day 7 and (**c**) day 3 and day 7, respectively.

The influence of Lepto-EVs on hBMSC general behavior was initially assessed by evaluating their uptake into recipient cells. CellMask™-labeled Lepto-EVs were successfully internalized by hBMSCs, with the labeled-EVs located within the cytoplasmic regions of the cell (Figure 5A). Treatment with Lepto-EVs significantly increased the hBMSC proliferative capacity over a 7-day period when compared to the untreated cells, assessed by quantifying metabolic activity and DNA content (Figure 5B and C) (*P* ≤ 0.01–0.0001). Additionally, hBMSC migration was substantially accelerated following Lepto-EV treatment, resulting in a 1.44-fold increase in % gap closure (58.72%) when compared to the untreated cells (40.70%) at 1 day (Figure 5D and E) (*P* ≤ 0.05).

### Therapeutic effects of Lepto-EVs within a cytokine-induced OA-like model

**Figure 6:**
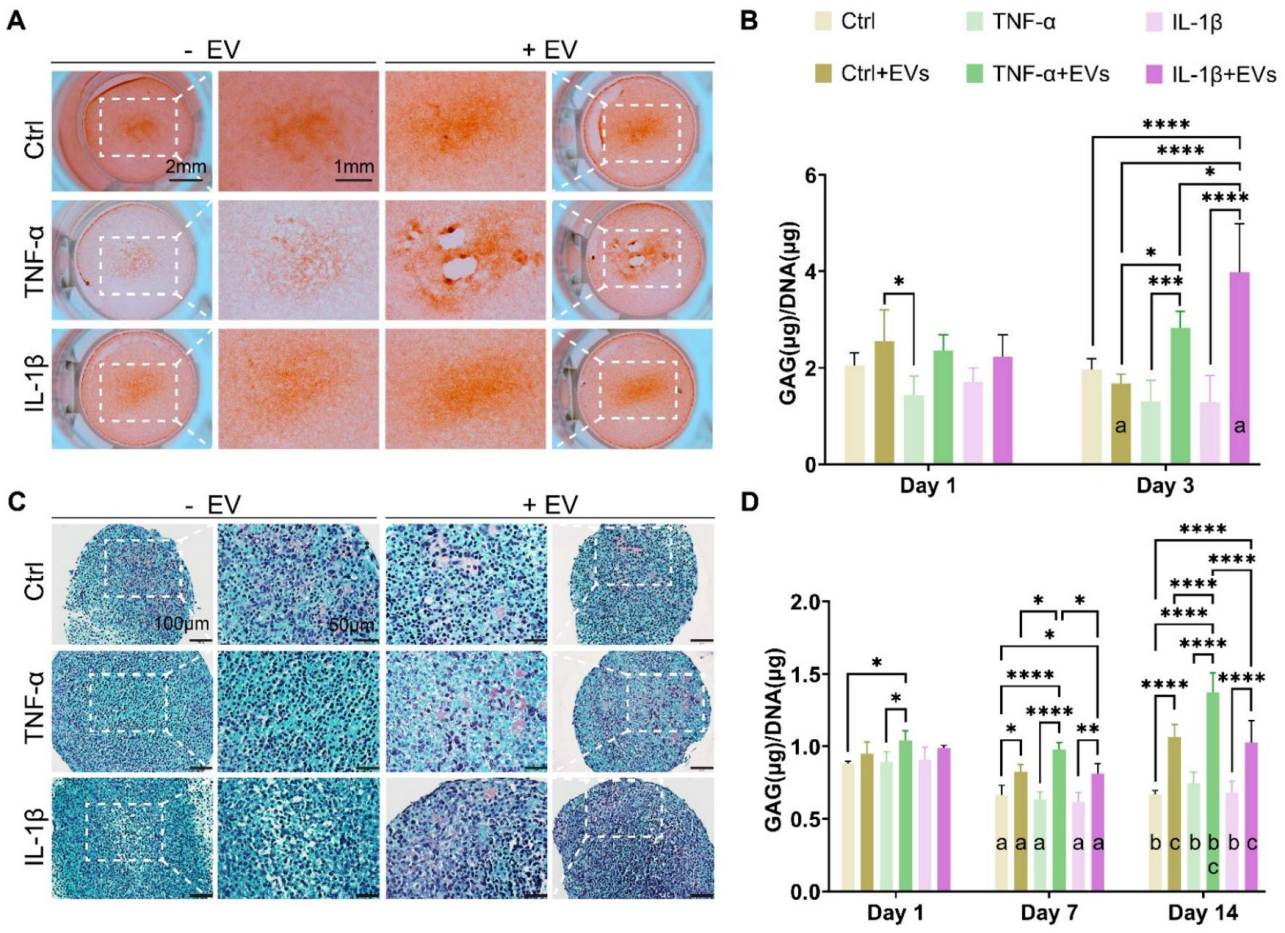
The effects of Lepto-EV treatment within a cytokine-induced OA-like *in vitro* model. (**A**) Safranin-O staining and (**B**) GAG content normalized by DNA content within a cytokine-induced 2D ATDC5 OA-like model (*n* ≥ 3). Letter (**a**) denotes significant differences (*P ≤ 0.05) between day 1 and day 3 within the same group. (**C**) Safranin O staining and (**D**) GAG content normalized by DNA content within a cytokine-induced 3D ATDC5 OA-like model (*n* = 5). Letters (**a**–**c**) denote significant differences (*P ≤ 0.05) within the same group between (**a**) day 1 and day 7, (**b**) day 1 and day 14, (**c**) day 7 and day 14, respectively. Data are expressed as mean ± SD. *P ≤ 0.05, **P ≤ 0.01, ***P ≤ 0.001 and ****P ≤ 0.0001.

We initially evaluated the antioxidant potential of Lepto-EVs in mitigating oxidative damage. Treatment with TNF-α and IL-1β elicited a significant increase in intracellular ROS levels in ATDC5 cells when compared to the untreated cells (*P* < 0.0001) (Supplementary Figure S6). In contrast, Lepto-EV treatment significantly reduced ROS production in the cells treated with TNF-α (28%) and IL-1β (30%) when compared to the cytokine alone group, restoring their oxidative status to basal levels. The effects of Lepto-EV treatment on alleviating cartilage-like matrix degradation were assessed using a cytokine-induced OA-like model. Within the 2D model, positive staining for GAGs was observed in all the treatment groups (Figure 6A). The TNF-α and IL-1β-treated groups exhibited reduced GAG staining intensity when compared to the untreated control at day 3. The Lepto-EV-treated cytokine groups exhibited a similar degree of GAG staining when compared to the untreated control. To further investigate the influence of Lepto-EV treatment on alleviating cytokine-induced cartilage-like matrix degradation, the quantification of GAG content was conducted (Figure 6B). Our findings showed a reduction in GAG content following TNF-α and IL-1β treatment when compared to the untreated control at both time points. Lepto-EV treatment did not improve GAG production in both the TNF-α and IL-1β treated groups at day 1 (*P* > 0.05), but improved GAG production at day 3 (*P* ≤ 0.001–0.0001) when compared to the EV-free cytokine-treated cells. An ATDC5-based 3D *in vitro* model [47, 48] was employed to further evaluate the influence of Lepto-EV treatment on reducing cytokine-induced matrix degradation within a more physiologically relevant system. Safranin-O staining was conducted to qualitatively assess GAG production. TNF-α and IL-1β treatment resulted in reduced Safranin-O staining intensity when compared to the cytokine-free control after 14 days of culture (Figure 6C). The Lepto-EV treated cytokine groups exhibited increased Safranin-O staining intensity when compared to the EV-free cytokine-treated groups. Following the quantification of GAG content, our findings showed that Lepto-EV treatment increased GAG production in the TNF-α and IL-1β-treated groups, as well as in the cytokine-free groups (Figure 6D).

## Discussion

Despite the growing interest in EVs as precision nanotherapeutics, their clinical translation remains constrained by fundamental limitations in mammalian cell-based production systems, including donor variability, low yield, labor-intensive isolation and scalability challenges [49, 50]. Here, we propose an alternative strategy using microalgae-derived EVs, leveraging these photosynthetic microorganisms’ unique capacity for scalable and economical production [37, 51]. We performed the first systematic optimization of microalgal EV production for potential therapeutic use, focusing on a clinically relevant OA disease model. Through comparative selection and iterative refinement of cultivation parameters, *Leptolyngbya* emerged as a lead species, demonstrating superior growth kinetics, cell viability and EV productivity under tailored photoperiod and harvesting conditions. Importantly, Lepto-EVs exhibited potent antioxidant, and anti-catabolic effects, underscoring their potential as the next-generation, cost-effective and sustainable EV-based nanotherapeutic for OA.

Our investigations into the biomanufacturing of microalgae EVs demonstrate the profound influence of cultivation conditions, particularly light exposure, on both algal growth and subsequent EV secretion. Our findings indicate that both microalgae growth and subsequent EV production are species- and light-dependent processes. For instance, *Leptolyngbya* demonstrated a clear advantage, showing superior growth kinetics and viability across different photoperiods, which directly correlated with a significantly higher total EV protein yield. This suggests that optimized growth conditions are a prerequisite for efficient EV production. In contrast, while *Chlorella* showed increased EV protein content with more light, its overall relatively lower viability and growth compared to other species present a challenge for its large-scale EV production efficiency. The unique response of *CC1690*, where increased light negatively impacted EV production despite high viability, underscores that the relationship between cellular health, growth and EV secretion is not always linear and is highly species-specific. The normalization of EV yield to microalgae number and biomass further refines this understanding, revealing that while *Leptolyngbya* is a top producer on a per-culture basis, *CC1690* may be a highly efficient producer on a per-cell basis. Beyond influencing microalgae growth and EV yield, our findings showcased that light exposure also plays a pivotal role in shaping the antioxidant capacity of Lepto-EVs. While continuous (24 h) illumination supported the highest growth rate and EV production, a 16 h light regimen markedly enhanced the antioxidant properties of the vesicles. Excessive light intensity likely compromises microalgae’s antioxidant capacity by inducing photoinhibition and excessive reactive oxygen species (ROS) generation, which can overwhelm cellular defence systems and degrade or suppress the synthesis of antioxidant molecules [52, 53]. Therefore, optimizing the light regime represents an effective strategy to fine-tune the therapeutic potential of Lepto-EVs, particularly for mitigating the excessive ROS associated with OA pathology.

In addition to light exposure, our results demonstrate the significant impact of medium harvesting frequency on EV production. Frequent medium replacement in microalgae cultures sustains nutrient availability, removes inhibitory metabolites and maintains stable pH and gas conditions, keeping algae in a metabolically active state [54]. This promotes faster growth and can enhance EV production, as vesicle release is linked to both cellular metabolism and transient stress responses [55]. By preventing nutrient limitation and self-inhibition while providing occasional mild stress cues, medium exchange effectively supports higher biomass accumulation and increased vesicle secretion. This approach contrasts with studies in the literature which typically relied on a single collection [56, 57], highlighting that our frequent collection strategy may contribute to sustained EV yields and more dynamic insights into vesicle production over time. These results are crucial for establishing a rational framework for microalgae-based EV manufacturing, confirming that selecting the appropriate species, optimizing light cycles and refining harvesting frequency are fundamental steps for maximizing the therapeutic potential of these nanovesicles.

Biophysical characterization confirmed that the obtained Lepto-EVs possess key hallmarks indicative of EVs, including their nanoscale size, spherical morphology and expression of classical EV-associated protein markers, such as CD63, EpCAM, ANXA5, TSG101, FLOT1, ICAM, ALIX and CD81, consistent with previous findings in the literature [56, 57]. It is known that many proteins are evolutionarily conserved across prokaryotes and eukaryotes, which can result in antibody recognition of homologous or structurally related proteins outside the species for which the antibodies were originally developed. Given the absence of universally accepted EV markers in cyanobacteria, these positive signals could be due to cross-reactivity with conserved EV protein motifs rather than canonical mammalian EV markers. Our findings showed that these Lepto-EVs were effectively internalized into the cytoplasm of recipient hBMSCs, demonstrating the inter-kingdom communication or cross-kingdom interaction between these plant-derived nanoparticles and human cells. Similar studies have demonstrated the internalization of EVs derived from *Spirulina platensis* (SP) [58] and *Tetraselmis chuii* [57] in ATDC5 and MDA-MB 231 cell lines, respectively.

The effective recruitment of endogenous cells is critical for subsequent tissue healing [59, 60]. Several studies have reported the capacity of EVs in stimulating the recruitment of progenitor cells in musculoskeletal applications [23, 61, 62]. Functionally, our findings showed that the Lepto-EVs were not only cytocompatible, but significantly enhanced the proliferation and migration capacity of progenitor cells, crucial cellular processes conducive to cartilage repair. This is consistent with evidence in the literature demonstrating the cytocompatibility of microalgae-derived EVs, although this has primarily been assessed with cell lines [56–58]. In recent years, there have been increasing studies investigating the xenogenic administration of human-, milk- and plant-derived EVs [63–65], particularly regarding their biosafety and biocompatibility. For instance, Adamo *et al*. administered *T. chuii*-derived EVs intravenously into immune-competent BALB/c mice and did not observe any noticeable local and systemic toxicity [57]. Similarly, Liang *et al*. reported that weekly intra-articular injections of SP-EVs in an OA mouse model caused no detectable adverse effects [58]. Our findings demonstrate the successful manufacture and isolation of Lepto-EVs, as well as their ability to interact and modulate the biological function of human-derived cells. These results underscore the potential of Lepto-EVs as a biologically active and biocompatible platform. Nonetheless, comprehensive safety assessments remain essential. Future studies should evaluate Lepto-EVs tolerability within *in vivo* studies, alongside detailed biodistribution information to fully define their therapeutic potential and translational applications.

To investigate the anti-catabolic effects of Lepto-EVs, a cytokine-induced OA-like *in vitro* model was employed as it provides a controlled and reproducible system to mimic the inflammatory and catabolic milieu of OA. This approach reduces donor variability, minimizes ethical and cost constraints and enables high-throughput assessment of EV bioactivity compared with *ex vivo* explants or *in vivo* models [66]. As such, it serves as an efficient first-line screening platform prior to validation in more complex tissue or animal models. Oxidative stress is a major driver of chondrocyte dysfunction and cartilage degeneration in OA. The ability of Lepto-EVs to reduce ROS production in cytokine-stimulated ATDC5 cells suggests that these vesicles can actively restore redox balance, consistent with studies in the literature [58, 67, 68]. This antioxidant effect may contribute to their broader chondroprotective and anti-inflammatory potential, supporting the use of microalgae-derived EVs as a therapeutic strategy to mitigate oxidative damage in OA. The cytokine-dependent reduction in GAG content during chondrogenic differentiation observed in both 2D and 3D models was consistent with findings of OA *in vitro* models in the literature [45, 69]. The findings from both 2D and 3D *in vitro* models provide evidence for the protective role of Lepto-EVs in mitigating cartilage-like matrix degradation associated with OA. In the 2D model, the reduction in Safranin-O staining and total GAG content following TNF-α and IL-1β stimulation was significantly countered by Lepto-EV treatment, with the GAG content returning to levels comparable to the untreated control. This suggests that Lepto-EVs can directly protect the cartilage-like extracellular matrix from inflammation-induced damage. The anti-catabolic effects of Lepto-EVs in our 2D ATDC5 model is consistent with the findings from Liang *et al*., who observed increased Sox9 and Col2a gene expression following SP-EV treatment of TNF-α stimulated ATDC5s [58]. The results from the 3D model, which more closely mimics the physiological environment of cartilage [70], further support this conclusion. The marked reduction in GAG content observed in the cytokine-treated groups was substantially reversed by Lepto-EVs, demonstrating their ability to preserve the integrity of the cartilage-like extracellular matrix, a crucial aspect of OA therapy [71, 72]. The observed increase in GAG content in cytokine-stimulated cells treated with Lepto-EVs highlights a reparative effect, suggesting that these vesicles may not only prevent matrix degradation, but may also promote anabolism within these chondrogenic cells. In this study, we initially used hBMSCs as a clinically relevant platform to evaluate EV internalization and general pro-regenerative cellular responses. The ATDC5 cells were employed as an OA-like model as they more closely exhibit OA-like chondrocyte phenotypes, which are not easily reproduced in hBMSC-derived cartilage constructs. This strategy allowed us to combine human translational relevance with disease-specific modeling. Although ATDC5s present a useful chondrogenic cell line [73, 74], it has been reported that the cartilage-like matrix produced from these cells is inherently less complex than that found in native articular cartilage [75]. Moreover, the high proliferative capacity of these cells rather than the stable, low-proliferative nature of native chondrocytes [76, 77], may overestimate effects on proliferative pathways while underrepresenting mechanisms relevant to quiescent or senescent chondrocytes. Additionally, it is important to note that ATDC5s are a teratocarcinoma-derived cell line, and they may respond differently to EV treatment compared to human chondrocytes. Nevertheless, the anti-catabolic findings observed in this study are likely mediated through the delivery of antioxidant and anti-inflammatory molecules inherent to the microalgal origin of the EVs [58, 78, 79].

To explore the potential bioactive cargo contributing to the antioxidant capacity of Lepto-EVs, we performed UV–Vis absorption spectroscopy. The spectral analysis revealed a prominent absorption peak at approximately 210 nm, which is commonly associated with the presence of phenolic and flavonoid compounds. These molecules are well known for their strong free-radical scavenging and anti-inflammatory properties [80]. Previous studies have reported that *Leptolyngbya* spp. are rich sources of phenolic and flavonoid metabolites [81], suggesting that these compounds may be selectively or passively packaged into Lepto-EVs under the cultivation conditions used in this study. In addition to phenolic and flavonoid metabolites, phycocyanin is another well-recognized bioactive molecule commonly found in cyanobacteria, including *Leptolyngbya*. However, our UV-Vis spectra did not show the characteristic phycocyanin absorption peak at approximately 620 nm, indicating that this pigment is likely not substantially incorporated into the vesicles under the current cultivation conditions. One possible explanation for this observation relates to differences in molecular size. Phenolic and flavonoid compounds typically have relatively low molecular weights (generally <1000 Da) [82, 83], whereas phycocyanin is a much larger protein complex with molecular weights ranging from approximately 10–21 kDa or higher [84, 85]. Such size differences may influence the efficiency with which these molecules are incorporated into EVs. Future studies employing multi-omics approaches, including proteomics, metabolomics and lipidomic, will be essential to comprehensively characterize the molecular cargo of Lepto-EVs and to better understand the components responsible for their observed bioactivity.

Our findings position Lepto-EVs as a potential next-generation cell-free nanotherapy for OA, with distinct advantages over existing biological approaches. While mammalian EVs can also carry bioactive cargo, their content is highly variable and donor-dependent [86, 87]. In contrast, microalgal EV composition can be more precisely modulated through environmental control, enabling a more standardized therapeutic product. Moreover, their environmental sustainability and ease of scale-up address key bottlenecks in the biomanufacturing pipeline, aligning with global priorities for green biotechnologies [37, 51]. However, several questions remain. The precise molecular cargo of Lepto-EVs, particularly their RNA and protein content, requires further elucidation to understand the mechanisms underlying their therapeutic effects. Additionally, while *in vitro* models provide important proof-of-concept data, *in vivo* studies are important to evaluate biodistribution, pharmacokinetics, immunogenicity and long-term efficacy. Prior to such *in vivo* studies, it is essential to develop EV-compatible biomaterial delivery systems that enable minimally invasive intra-articular administration and controlled release, reducing the frequency of injections and associated animal stress. Employing *in vivo* models that replicate the diverse endotypes of OA is important to gain mechanistic insight, ensures translational relevance and minimizes model-specific bias. The present findings establish a robust platform for Lepto-EV production, characterization and preliminary functional evaluation, thereby providing a strong foundation for future preclinical testing. Direct comparison of Lepto-EVs with mammalian-derived EVs (e.g. MSC-EVs) in these models would further clarify the potential of microalgae as a novel source of therapeutic vesicles and inform translational development for OA treatment.

Importantly, regulatory pathways for algae-derived nanotherapeutics remain underdeveloped, necessitating the dialogue with policymakers and standardization bodies. The clinical translation of plant- and microalgae-derived EVs remains in its early stages, largely due to regulatory and classification challenges. For pharmaceutical applications, mammalian-based EV products may be classified as biological medicinal products, requiring precise definition of active substances and elucidation of mechanisms of action [88]. In contrast, plant-derived EV-based products may fall under the category of botanical drugs, for which demonstrating safety and efficacy is sufficient under FDA guidance [89]. In the EU, plant-derived EV products could be regulated as advanced therapy medicinal products (ATMPs), necessitating thorough evaluation of pharmacological properties, manufacturing processes and quality control. Comprehensive physicochemical profiling, safety validation and standardized manufacturing are therefore essential to meet these regulatory demands and advance the clinical and commercial translation of plant-derived nanovesicles.

In addition to demonstrating potent regenerative effects *in vitro*, the present study underscores the economic and biomanufacturing potential of microalgae-derived EVs as next-generation nanotherapeutics. Unlike MSC-derived EVs, which are hampered by costly xeno-free media, and limited scalability [90], microalgal platforms can be cultivated in photobioreactors with minimal nutrient inputs and reliance on light and CO_2_, thereby markedly reducing upstream costs and reducing the high carbon footprint associated with mammalian cell manufacture [91, 92]. Although downstream purification and regulatory frameworks remain to be fully optimized, the combination of rapid biomass accumulation, high EV yield and sustainable (lower carbon footprint) production positions Lepto-EVs as a viable alternative to EVs derived from mammalian sources. By bridging nanomedicine, environmental biotechnology and regenerative medicine, our findings advance microalgae EVs as a scalable, cost-efficient and environmentally sustainable nanotherapeutic platform with significant translational potential in regenerative medicine.

## Conclusion

In conclusion, this study demonstrates that microalgae can serve as a sustainable, non-mammalian platform for the scalable production of EVs with therapeutic potential for OA. Through systematic species selection and controlled photoperiod conditions, we identified *Leptolyngbya*-derived EVs (Lepto-EVs) as a highly promising nanotherapeutic candidate, exhibiting superior production efficiency, pronounced antioxidant activity and robust anti-catabolic effects compared to EVs from other microalgal or mammalian sources reported in previous studies. This work introduces the novel concept that environmental regulation of EV biogenesis can be harnessed to tune nanoscale properties and bioactivity, bridging a critical gap between colloid interface science and nanomedicine. Compared with prior EV production strategies, our approach improves scalability, reduces batch variability, and leverages an environmentally sustainable platform, representing a significant advance toward clinically translatable, cell-free therapeutics. Looking forward, these findings provide a framework for engineering algae-derived EVs with tailored molecular cargos and physicochemical properties, opening avenues for next-generation nanotherapeutics targeting OA and other degenerative diseases.

## Supplementary Material

rbag074_Supplementary_Data

## Data Availability

The datasets used and/or analysed during the current study are available from the corresponding author on reasonable request.

## References

[1] Loeser RF , Goldring SR, Scanzello CR, Goldring MB. Osteoarthritis: a disease of the joint as an organ. Arthritis Rheum 2012;64:1697–707. 22392533 10.1002/art.34453 PMC3366018

[2] Runge N , Aina A, May S. The benefits of adding manual therapy to exercise therapy for improving pain and function in patients with knee or hip osteoarthritis: a systematic review with meta-analysis. J Orthop Sports Phys Ther 2022;52:675–A13. 35881705 10.2519/jospt.2022.11062

[3] Steinmetz JD , Culbreth GT, Haile LM, Rafferty Q, Lo J, Fukutaki KG, Cruz JA, Smith AE, Vollset SE, Brooks PM, et al Global, regional, and national burden of osteoarthritis, 1990–2020 and projections to 2050: a systematic analysis for the global burden of disease study 2021. Lancet Rheumatol 2023;5:e508–22. 37675071 10.1016/S2665-9913(23)00163-7 PMC10477960

[4] Leifer VP , Katz JN, Losina E. The burden of OA-health services and economics. Osteoarthritis Cartilage 2022;30:10–6. 34023527 10.1016/j.joca.2021.05.007 PMC8605034

[5] Lo J , Chan L, Flynn S. A systematic review of the incidence, prevalence, costs, and activity and work limitations of amputation, osteoarthritis, rheumatoid arthritis, back pain, multiple sclerosis, spinal cord injury, stroke, and traumatic brain injury in the United States: a 2019 update. Arch Phys Med Rehabil 2021;102:115–31. 32339483 10.1016/j.apmr.2020.04.001 PMC8529643

[6] Chen D , Shen J, Zhao W, Wang T, Han L, Hamilton JL, Im H-J. Osteoarthritis: toward a comprehensive understanding of pathological mechanism. Bone Res 2017;5:16044. 28149655 10.1038/boneres.2016.44 PMC5240031

[7] Gil Izquierdo S , Fernández Pilar A, Rios JL, Lim KS, Toh WS, Liu C, Gimona M, Gawlitta D, Man K. Advances in extracellular vesicle-based nanomedicine for regenerative orthopaedics. J Nanobiotechnology 2025;24:36. 41392147 10.1186/s12951-025-03906-w PMC12797946

[8] Figueroa-Valdés AI , Luz-Crawford P, Herrera-Luna Y, Georges-Calderón N, García C, Tobar HE, Araya MJ, Matas J, Donoso-Meneses D, de la Fuente C, et al Clinical-grade extracellular vesicles derived from umbilical cord mesenchymal stromal cells: preclinical development and first-in-human intra-articular validation as therapeutics for knee osteoarthritis. J Nanobiotechnology 2025;23:13. 39806427 10.1186/s12951-024-03088-x PMC11730155

[9] Cong B , Sun T, Zhao Y, Chen M. Current and novel therapeutics for articular cartilage repair and regeneration. Ther Clin Risk Manag 2023;19:485–502. 37360195 10.2147/TCRM.S410277 PMC10290456

[10] Kloppenburg M , Berenbaum F. Osteoarthritis year in review 2019: epidemiology and therapy. Osteoarthritis Cartilage 2020;28:242–8. 31945457 10.1016/j.joca.2020.01.002

[11] Chow YY , Chin K-Y. The role of inflammation in the pathogenesis of osteoarthritis. Mediators Inflamm 2020;2020:8293921. 32189997 10.1155/2020/8293921 PMC7072120

[12] Ansari MY , Ahmad N, Haqqi TM. Oxidative stress and inflammation in osteoarthritis pathogenesis: role of polyphenols. Biomed Pharmacother 2020;129:110452. 32768946 10.1016/j.biopha.2020.110452 PMC8404686

[13] Hwang H , Kim H. Chondrocyte apoptosis in the pathogenesis of osteoarthritis. Int J Mol Sci 2015;16:26035–54. 26528972 10.3390/ijms161125943 PMC4661802

[14] Wei G , Lu K, Umar M, Zhu Z, Lu WW, Speakman JR, Chen Y, Tong L, Chen D. Risk of metabolic abnormalities in osteoarthritis: a new perspective to understand its pathological mechanisms. Bone Res 2023;11:63. 38052778 10.1038/s41413-023-00301-9 PMC10698167

[15] Zhidu S , Ying T, Rui J, Chao Z. Translational potential of mesenchymal stem cells in regenerative therapies for human diseases: challenges and opportunities. Stem Cell Res Ther 2024;15:266. 39183341 10.1186/s13287-024-03885-z PMC11346273

[16] Kangari P , Talaei-Khozani T, Razeghian-Jahromi I, Razmkhah M. Mesenchymal stem cells: amazing remedies for bone and cartilage defects. Stem Cell Res Ther 2020;11:492. 33225992 10.1186/s13287-020-02001-1 PMC7681994

[17] Marei HE. Stem cell therapy: a revolutionary cure or a pandora’s box. Stem Cell Res Ther 2025;16:255. 40405306 10.1186/s13287-025-04334-1 PMC12096755

[18] Hoang DM , Pham PT, Bach TQ, Ngo ATL, Nguyen QT, Phan TTK, Nguyen GH, Le PTT, Hoang VT, Forsyth NR, Heke M, Nguyen LT. Stem cell-based therapy for human diseases. Signal Transduct Target Ther 2022;7:272. 35933430 10.1038/s41392-022-01134-4 PMC9357075

[19] Ebrahimi F , Kumari A, Ghadami S, Al Abdullah S, Dellinger K. The potential for extracellular vesicles in nanomedicine: a review of recent advancements and challenges ahead. Adv Biol 2025;9. 10.1002/adbi.202400623 PMC12206938 39739455

[20] Man K , Brunet MY, Jones M-C, Cox SC. Engineered extracellular vesicles: tailored-made nanomaterials for medical applications. Nanomaterials 2020;10:1838. 32942556 10.3390/nano10091838 PMC7558114

[21] Pol F , Longoni A, Levato R, Gawlitta D, Man K. Extracellular vesicles in osteoimmunomodulation: orchestrating immune-driven bone regeneration. Int J Biol Macromol 2026;338:149614. 41391798 10.1016/j.ijbiomac.2025.149614

[22] Raposo G , Stoorvogel W. Extracellular vesicles: exosomes, microvesicles, and friends. J Cell Biol 2013;200:373–83. 23420871 10.1083/jcb.201211138 PMC3575529

[23] Staubli F , Sobrevals Alcaraz P, van Es RM, Vos HR, Bergsma E, Gawlitta D, Man K. Bioengineering developmentally inspired matrix vesicles as designer nanotherapeutics for bone regeneration. *Regen. Biomater *2026;rbag075. 10.1093/rb/rbag075 PMC13198379 42179884

[24] Unnithan AR , Man K, Gethings LA, Hughes CJ, Keenan A, Heaney L, Cox SC, Davies OG, El Haj AJ, Kritika. Engineering extracellular vesicle production through magnetic ion channel activation for bone regeneration. Adv Healthc Mater 2026;e04542. 41797444 10.1002/adhm.202504542 PMC13176524

[25] Man K , Eisenstein NM, Hoey DA, Cox SC. Bioengineering extracellular vesicles: smart nanomaterials for bone regeneration. J Nanobiotechnology 2023;21:137. 37106449 10.1186/s12951-023-01895-2 PMC10134574

[26] Feng K , Wang F, Chen H, Zhang R, Liu J, Li X, Xie X, Kang Q. Cartilage progenitor cells derived extracellular vesicles-based cell-free strategy for osteoarthritis treatment by efficient inflammation inhibition and extracellular matrix homeostasis restoration. J Nanobiotechnology 2024;22:345. 38890638 10.1186/s12951-024-02632-z PMC11186174

[27] Zhang B , Tian X, Qu Z, Hao J, Zhang W. Hypoxia-preconditioned extracellular vesicles from mesenchymal stem cells improve cartilage repair in osteoarthritis. Membranes (Basel) 2022;12. 10.3390/membranes12020225 PMC8875566 35207146

[28] Du S , Guan Y, Xie A, Yan Z, Gao S, Li W, Rao L, Chen X, Chen T. Extracellular vesicles: a rising star for therapeutics and drug delivery. J Nanobiotechnology 2023;21:231. 37475025 10.1186/s12951-023-01973-5 PMC10360328

[29] Ha D , Yang N, Nadithe V. Exosomes as therapeutic drug carriers and delivery vehicles across biological membranes: current perspectives and future challenges. Acta Pharm Sin B 2016;6:287–96. 27471669 10.1016/j.apsb.2016.02.001 PMC4951582

[30] Silva RM , Rosa SS, Santos JAL, Azevedo AM, Fernandes‐Platzgummer A. Enabling mesenchymal stromal cells and their extracellular vesicles clinical availability—a technological and economical evaluation. J Extracell Biol 2025;4:e70037. 40104174 10.1002/jex2.70037 PMC11913891

[31] Tan F , Li X, Wang Z, Li J, Shahzad K, Zheng J. Clinical applications of stem cell-derived exosomes. Signal Transduct Target Ther 2024;9:17. 38212307 10.1038/s41392-023-01704-0 PMC10784577

[32] Staubli F , Zhou Y, Vader P, Hofmann S, Bergsma E, Gawlitta D, Man K. Harnessing extracellular vesicles for endochondral bone regeneration: mechanisms and applications. Acta Biomater 2026;212:168–86. 41519364 10.1016/j.actbio.2026.01.012

[33] Khan MI , Shin JH, Kim JD. The promising future of microalgae: current status, challenges, and optimization of a sustainable and renewable industry for biofuels, feed, and other products. Microb Cell Fact 2018;17:36. 29506528 10.1186/s12934-018-0879-x PMC5836383

[34] Spolaore P , Joannis-Cassan C, Duran E, Isambert A. Commercial applications of microalgae. J Biosci Bioeng 2006;101:87–96. 16569602 10.1263/jbb.101.87

[35] Dolganyuk V , Belova D, Babich O, Prosekov A, Ivanova S, Katserov D, Patyukov N, Sukhikh S. Microalgae: a promising source of valuable bioproducts. Biomolecules 2020;10:1153. 32781745 10.3390/biom10081153 PMC7465300

[36] Taunt HN , Stoffels L, Purton S. Green biologics: the algal chloroplast as a platform for making biopharmaceuticals. Bioengineered 2018;9:48–54. 28892417 10.1080/21655979.2017.1377867 PMC5972929

[37] Einhaus A , Baier T, Kruse O. Molecular design of microalgae as sustainable cell factories. Trends Biotechnol 2024;42:728–38. 38092627 10.1016/j.tibtech.2023.11.010

[38] Kholssi R , Lougraimzi H, Moreno-Garrido I. Effects of global environmental change on microalgal photosynthesis, growth and their distribution. Mar Environ Res 2023;184:105877. 36640723 10.1016/j.marenvres.2023.105877

[39] Usher PK , Ross AB, Camargo-Valero MA, Tomlin AS, Gale WF. An overview of the potential environmental impacts of large-scale microalgae cultivation. Biofuels 2014;5:331–49.

[40] Brand-Williams W , Cuvelier ME, Berset C. Use of a free radical method to evaluate antioxidant activity. LWT – Food Sci Technol 1995;28:25–30.

[41] Kilasoniya A , Garaeva L, Shtam T, Spitsyna A, Putevich E, Moreno-Chamba B, Salazar-Bermeo J, Komarova E, Malek A, Valero M, Saura D. Potential of plant exosome vesicles from grapefruit (*Citrus × paradisi*) and tomato (*Solanum lycopersicum*) juices as functional ingredients and targeted drug delivery vehicles. Antioxidants 2023;12. 10.3390/antiox12040943 PMC10135875 37107317

[42] Gawlitta D , van Rijen MHP, Schrijver EJM, Alblas J, Dhert WJA. Hypoxia impedes hypertrophic chondrogenesis of human multipotent stromal cells. Tissue Eng Part A 2012;18:1957–66. 22563686 10.1089/ten.TEA.2011.0657

[43] Man K , Brunet MY, Louth S, Robinson TE, Fernandez-Rhodes M, Williams S, Federici AS, Davies OG, Hoey DA, Cox SC. Development of a bone-mimetic 3D printed Ti6Al4V scaffold to enhance osteoblast-derived extracellular vesicles’ therapeutic efficacy for bone regeneration. Front Bioeng Biotechnol 2021;9:757220. 34765595 10.3389/fbioe.2021.757220 PMC8576375

[44] Man K , Brunet MY, Fernandez-Rhodes M, Williams S, Heaney LM, Gethings LA, Federici A, Davies OG, Hoey D, Cox SC. Epigenetic reprogramming enhances the therapeutic efficacy of osteoblast-derived extracellular vesicles to promote human bone marrow stem cell osteogenic differentiation. J Extracell Vesicles 2021;10:e12118. 34262674 10.1002/jev2.12118 PMC8263905

[45] Wehling N , Palmer GD, Pilapil C, Liu F, Wells JW, Müller PE, Evans CH, Porter RM. Interleukin‐1β and tumor necrosis factor α inhibit chondrogenesis by human mesenchymal stem cells through NF‐κB-dependent pathways. Arthritis Rheum 2009;60:801–12. 19248089 10.1002/art.24352 PMC2688727

[46] Ruhl T , Beier JP. Quantification of chondrogenic differentiation in monolayer cultures of mesenchymal stromal cells. Anal Biochem 2019;582:113356. 31276649 10.1016/j.ab.2019.113356

[47] Wu X , Zhao K, Fang X, Lu F, Cheng P, Song X, Zhang W, Yao C, Zhu J, Chen H. Saikosaponin D inhibited IL-1β induced ATDC 5 chondrocytes apoptosis In vitro and delayed articular cartilage degeneration in OA model mice In vivo. Front Pharmacol 2022;13:845959. 35370642 10.3389/fphar.2022.845959 PMC8975252

[48] Yang X , Zhou Y, Chen Z, Chen C, Han C, Li X, Tian H, Cheng X, Zhang K, Zhou T, Zhao J. Curcumenol mitigates chondrocyte inflammation by inhibiting the NF-κB and MAPK pathways, and ameliorates DMM-induced OA in mice. Int J Mol Med 2021;48:192. 34435650 10.3892/ijmm.2021.5025 PMC8416138

[49] Liu D , Gao J, Wu X, Han L. Plant-derived exosome-like nanoparticles as promising biotherapeutic tools: recent advances and challenges. Smart Mater Med 2025;6:285–304.

[50] Welsh JA , Goberdhan DCI, O’Driscoll L, Buzas EI, Blenkiron C, Bussolati B, Cai H, Di Vizio D, Driedonks TAP, Erdbrügger U, et al MISEV Consortium. Minimal information for studies of extracellular vesicles (MISEV2023): from basic to advanced approaches. J Extracell Vesicles 2024;13:e12404. 38326288 10.1002/jev2.12404 PMC10850029

[51] Onyeaka H , Miri T, Obileke K, Hart A, Anumudu C, Al-Sharify ZT. Minimizing carbon footprint via microalgae as a biological capture. Carbon Capture Sci Technol 2021;1:100007.

[52] Coulombier N , Nicolau E, Le Déan L, Antheaume C, Jauffrais T, Lebouvier N. Impact of light intensity on antioxidant activity of tropical microalgae. Mar Drugs 2020;18:122. 32085557 10.3390/md18020122 PMC7073765

[53] Songserm R , Nishiyama Y, Sanevas N. Light influences the growth, pigment synthesis, photosynthesis capacity, and antioxidant activities in *Scenedesmus falcatus*. Scientifica (Cairo) 2024;2024:1898624–12. 38293704 10.1155/2024/1898624 PMC10827371

[54] Sharma AK , Jaryal S, Sharma S, Dhyani A, Tewari BS, Mahato N. Biofuels from microalgae: a review on microalgae cultivation, biodiesel production techniques and storage stability. Processes 2025;13:488.

[55] Gebremedhn S , Ali A, Gad A, Prochazka R, Tesfaye D. Extracellular vesicles as mediators of environmental and metabolic stress coping mechanisms during mammalian follicular development. Front Vet Sci 2020;7:602043. 33330723 10.3389/fvets.2020.602043 PMC7710682

[56] Adamo G , Fierli D, Romancino DP, Picciotto S, Barone ME, Aranyos A, Božič D, Morsbach S, Raccosta S, Stanly C, et al Nanoalgosomes: introducing extracellular vesicles produced by microalgae. J Extracell Vesicles 2021;10:e12081. 33936568 10.1002/jev2.12081 PMC8077145

[57] Adamo G , Santonicola P, Picciotto S, Gargano P, Nicosia A, Longo V, Aloi N, Romancino DP, Paterna A, Rao E, et al Extracellular vesicles from the microalga *Tetraselmis chuii* are biocompatible and exhibit unique bone tropism along with antioxidant and anti-inflammatory properties. Commun Biol 2024;7:941. 39097626 10.1038/s42003-024-06612-9 PMC11297973

[58] Liang F , Zheng Y, Zhao C, Li L, Hu Y, Wang C, Wang R, Feng T, Liu X, Cui J, Zhong D, Zhou M. Microalgae-derived extracellular vesicles synergize with herbal hydrogel for energy homeostasis in osteoarthritis treatment. ACS Nano 2025;19:8040–57. 39982764 10.1021/acsnano.4c16085

[59] Pacelli S , Basu S, Whitlow J, Chakravarti A, Acosta F, Varshney A, Modaresi S, Berkland C, Paul A. Strategies to develop endogenous stem cell-recruiting bioactive materials for tissue repair and regeneration. Adv Drug Deliv Rev 2017;120:50–70. 28734899 10.1016/j.addr.2017.07.011 PMC5705585

[60] Yang Z , Li H, Yuan Z, Fu L, Jiang S, Gao C, Wang F, Zha K, Tian G, Sun Z, Huang B, Wei F, Cao F, Sui X, Peng J, Lu S, Guo W, Liu S, Guo Q. Endogenous cell recruitment strategy for articular cartilage regeneration. Acta Biomater 2020;114:31–52. 32652223 10.1016/j.actbio.2020.07.008

[61] Man K , Barroso IA, Brunet MY, Peacock B, Federici AS, Hoey DA, Cox SC. Controlled release of epigenetically-enhanced extracellular vesicles from a GelMA/nanoclay composite hydrogel to promote bone repair. Int J Mol Sci 2022;23:832. 35055017 10.3390/ijms23020832 PMC8775793

[62] Mao G , Zhang Z, Hu S, Zhang Z, Chang Z, Huang Z, Liao W, Kang Y. Exosomes derived from miR-92a-3p-overexpressing human mesenchymal stem cells enhance chondrogenesis and suppress cartilage degradation via targeting WNT5A. Stem Cell Res Ther 2018;9:247. 30257711 10.1186/s13287-018-1004-0 PMC6158854

[63] Somiya M , Yoshioka Y, Ochiya T. Biocompatibility of highly purified bovine milk‐derived extracellular vesicles. J Extracell Vesicles 2018;7:1440132. 29511463 10.1080/20013078.2018.1440132 PMC5827637

[64] Forteza-Genestra MA , Antich-Rosselló M, Ráez-Meseguer C, Sangenís AT, Calvo J, Gayà A, Monjo M, Ramis JM. Intra-articular injection of platelet lysate-derived extracellular vesicles recovers from knee osteoarthritis in an in vivo rat model. J Orthop Translat 2024;45:1–9. 38371711 10.1016/j.jot.2023.10.005 PMC10873568

[65] Weng J , Chen Y, Zeng Y, Jin W, Ji Y, Zhang W, Wang S, Li H, Yi M, Niu X, Deng X, Huang J, Su X, Chen L. A novel hydrogel loaded with plant exosomes and stem cell exosomes as a new strategy for treating diabetic wounds. Mater Today Bio 2025;32:101810. 10.1016/j.mtbio.2025.101810 PMC12088786 40391025

[66] Scalzone A , Cerqueni G, Wang XN, Dalgarno K, Mattioli-Belmonte M, Ferreira-Duarte AM, Gentile P. A cytokine-induced spheroid-based in vitro model for studying osteoarthritis pathogenesis. Front Bioeng Biotechnol 2023;11:1167623. 37229489 10.3389/fbioe.2023.1167623 PMC10203413

[67] Koh J-A , Le MH, Lee H-J, Min J-W, Kim MJ, Kang H-C, Kim Y-J. Extracellular vesicles derived from *Lactobacillus gasseri* GFC-1220 alleviate inflammation via the TLR4/NF-κB signaling pathway in LPS-stimulated RAW264.7 macrophages. Sci Rep 2025;15:21381. 40595215 10.1038/s41598-025-99160-z PMC12215889

[68] Gargano P , Picciotto S, Paterna A, Raccosta S, Rao E, Romancino DP, Smeraldi G, Manno M, Salamone M, Zarovni N, Adamo G, Bongiovanni A. Extracellular vesicles from microalgae as natural bioactive agents for UVB protection, anti-aging, and skin depigmentation. Biomed Pharmacother 2026;194:118961. 41496353 10.1016/j.biopha.2025.118961

[69] Zhou J , Wang Q. Daphnoretin relieves IL-1β-mediated chondrocytes apoptosis via repressing endoplasmic reticulum stress and NLRP3 inflammasome. J Orthop Surg Res 2022;17:487. 36384642 10.1186/s13018-022-03316-w PMC9670399

[70] Samvelyan HJ , Hughes D, Stevens C, Staines KA. Models of osteoarthritis: relevance and new insights. Calcif Tissue Int 2021;109:243–56. 32062692 10.1007/s00223-020-00670-x PMC8403120

[71] Li S , Cao P, Chen T, Ding C. Latest insights in disease-modifying osteoarthritis drugs development. Ther Adv Musculoskelet Dis 2023;15:1759720X231169839. 10.1177/1759720X231169839 PMC10184265 37197024

[72] Rapp AE , Zaucke F. Cartilage extracellular matrix-derived matrikines in osteoarthritis. Am J Physiol-Cell Physiol 2023;324:C377–94. 36571440 10.1152/ajpcell.00464.2022

[73] Yao Y , Wang Y. ATDC5: an excellent in vitro model cell line for skeletal development. J Cell Biochem 2013;114:1223–9. 23192741 10.1002/jcb.24467

[74] Xu R , Wei Y, Yin X, Shi B, Li J. miR-20a suppresses chondrogenic differentiation of ATDC5 cells by regulating Atg7. Sci Rep 2019;9:9243. 31239522 10.1038/s41598-019-45502-7 PMC6592888

[75] Wilhelm D , Kempf H, Bianchi A, Vincourt J-B. ATDC5 cells as a model of cartilage extracellular matrix neosynthesis, maturation and assembly. J Proteomics 2020;219:103718. 32097723 10.1016/j.jprot.2020.103718

[76] Newton PT , Staines KA, Spevak L, Boskey AL, Teixeira CC, Macrae VE, Canfield AE, Farquharson C. Chondrogenic ATDC5 cells: an optimised model for rapid and physiological matrix mineralisation. Int J Mol Med 2012;30:1187–93. 22941229 10.3892/ijmm.2012.1114 PMC3573767

[77] Phull A-R , Eo S-H, Abbas Q, Ahmed M, Kim SJ. Applications of chondrocyte-based cartilage engineering: an overview. Biomed Res Int 2016;2016:1879837–17. 27631002 10.1155/2016/1879837 PMC5007317

[78] Usui K , Yamamoto H, Mori H, Fujita Y. Extracellular vesicle-mediated secretion of chlorophyll biosynthetic intermediates in the cyanobacterium *Leptolyngbya boryana*. *Plant Cell Physiol ***2025**;66:214–28. 10.1093/pcp/pcae095 PMC11879085 39172638

[79] Maltsev Y , Maltseva K, Kulikovskiy M, Maltseva S. Influence of light conditions on microalgae growth and content of lipids, carotenoids, and fatty acid composition. Biology (Basel) 2021;10. 10.3390/biology10101060 PMC8533579 34681157

[80] Yılmazer Keskin S , Avcı A, Fajriana Febda Kurnia H. Analyses of phytochemical compounds in the flowers and leaves of *Spiraea japonica* var. *fortunei* using UV–VIS, FTIR, and LC–MS techniques. Heliyon 2024;10:e25496. 38327478 10.1016/j.heliyon.2024.e25496 PMC10848007

[81] Ijaz S , Hasnain S. Antioxidant potential of indigenous cyanobacterial strains in relation with their phenolic and flavonoid contents. Nat Prod Res 2016;30:1297–300. 26150139 10.1080/14786419.2015.1053088

[82] Gates PJ , Lopes NP. Characterisation of flavonoid aglycones by negative ion chip-based nanospray tandem mass spectrometry. Int J Anal Chem 2012;2012:259217 22505914 10.1155/2012/259217 PMC3296263

[83] Carregosa D , Carecho R, Figueira I, N Santos C. Low-molecular weight metabolites from polyphenols as effectors for attenuating neuroinflammation. J Agric Food Chem 2020;68:1790–807. 31241945 10.1021/acs.jafc.9b02155

[84] Kathiravan A , Udayan E, Ranjith Kumar R. Bioprospecting of spirulina biomass using novel extraction method for the production of C-phycocyanin as effective food colourant. Vegetos 2022;35:484–92.

[85] Shafiei M , Shafiei M, Mohseni Sani N, Guo W, Guo S, Vali H, Akbari Noghabi K. A new and promising C-phycocyanin-producing cyanobacterial strain, *Cyanobium* sp. MMK01: practical strategy towards developing a methodology to achieve C-phycocyanin with ultra-high purity. Front Microbiol 2024;15:1394617. 39640854 10.3389/fmicb.2024.1394617 PMC11617521

[86] O’Brien K , Breyne K, Ughetto S, Laurent LC, Breakefield XO. RNA delivery by extracellular vesicles in mammalian cells and its applications. Nat Rev Mol Cell Biol 2020;21:585–606. 32457507 10.1038/s41580-020-0251-y PMC7249041

[87] Mohamed AH , Abaza T, Youssef YA, Rady M, Fahmy SA, Kamel R, Hamdi N, Efthimiado E, Braoudaki M, Youness RA. Extracellular vesicles: from intracellular trafficking molecules to fully fortified delivery vehicles for cancer therapeutics. Nanoscale Adv 2025;7:934–62. 39823046 10.1039/d4na00393d PMC11733735

[88] Lener T , Gimona M, Aigner L, Börger V, Buzas E, Camussi G, Chaput N, Chatterjee D, Court FA, Del Portillo HA, et al Applying extracellular vesicles based therapeutics in clinical trials—an ISEV position paper. J Extracell Vesicles 2015;4:30087. 26725829 10.3402/jev.v4.30087 PMC4698466

[89] Jo HY , Kang SJ, Kim G, Gwak S, Baek G, Rhee WJ. Plant‐derived extracellular vesicles: current status and challenges for developing a new paradigm in therapeutics development. VIEW 2025;6.

[90] Estes S , Konstantinov K, Young JD. Manufactured extracellular vesicles as human therapeutics: challenges, advances, and opportunities. Curr Opin Biotechnol 2022;77:102776. 36041354 10.1016/j.copbio.2022.102776

[91] Paterna A , Rao E, Adamo G, Raccosta S, Picciotto S, Romancino D, Noto R, Touzet N, Bongiovanni A, Manno M. Isolation of extracellular vesicles from microalgae: a renewable and scalable bioprocess. Front Bioeng Biotechnol 2022;10:836747. 35360396 10.3389/fbioe.2022.836747 PMC8963918

[92] Mehariya S , Goswami RK, Karthikeysan OP, Verma P. Microalgae for high-value products: a way towards green nutraceutical and pharmaceutical compounds. Chemosphere 2021;280:130553. 33940454 10.1016/j.chemosphere.2021.130553

