# Supplementary material for: Refining the biomanufacturing of microalgae-derived extracellular vesicles as a potential nanotherapeutic for osteoarthritis
Source: Regen Biomater. 2026 Apr 16;13:rbag074. doi: 10.1093/rb/rbag074 (PMC13178680; doi:10.1093/rb/rbag074)
Supplement: rbag074_Supplementary_Data [file rbag074_supplementary_data.docx]

**Supplementary materials**

**Supplementary Table 1: Microalgal cultivation media**

**Supplementary Table 2: Formula used for calculating microalgae cell number. DC: dilute coefficient**

| Microalgal species | Cell counting (*10^6^ cells ml^-1^) | R^2^ |
| --- | --- | --- |
| *Chlorella sorokiniana* | 18.181*OD750*2.9*DC + 58.552 | 0.9367 |
| *Synechococcus* sp. | 107.020*OD750*2.74*DC + 8.049 | 0.9132 |
| *Leptolyngbya* sp. | (27.421*OD750*2.21*DC*100 - 59.365)/100 | 0.8713 |
| *Chlamydomonas reinhardtii CC1690* | 4.008*OD750*1.29*DC + 1.407 | 0.953 |

**Supplementary Table 3: Formula used for calculating microalgal dry biomass. DC: dilute coefficient**

| Microalgal species | Biomass counting (*g L^-1^) | R^2^ |
| --- | --- | --- |
| *Chlorella sorokiniana* | 0.3354*OD750*2.9*DC -0.3868 | 0.937 |
| *Synechococcus* sp. | 0.334*OD750*2.74*DC + 0.2087 | 0.913 |
| *Leptolyngbya* sp. | (0.4672*OD750*2.21*DC - 0.2422) | 0.956 |
| *Chlamydomonas reinhardtii CC1690* | 0.657*OD750*1.29*DC + 0.031 | 0.99 |


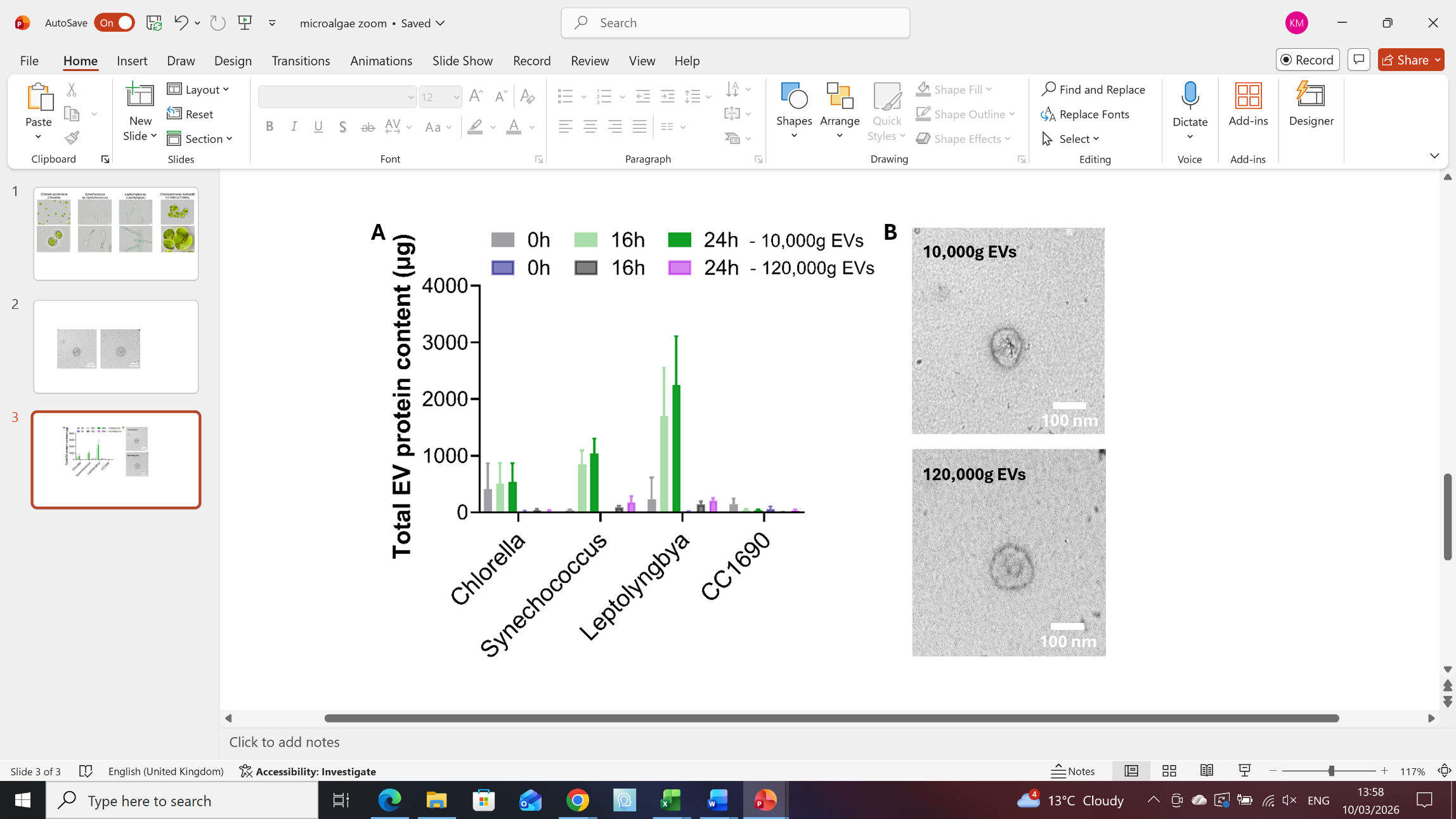


**Supplementary Figure 1. The effect of microalgae isolation on EV yield and morphology.** A) Quantification of EV production (total EV protein content), B) TEM analysis of isolated EVs. Data expressed as mean ± SD.


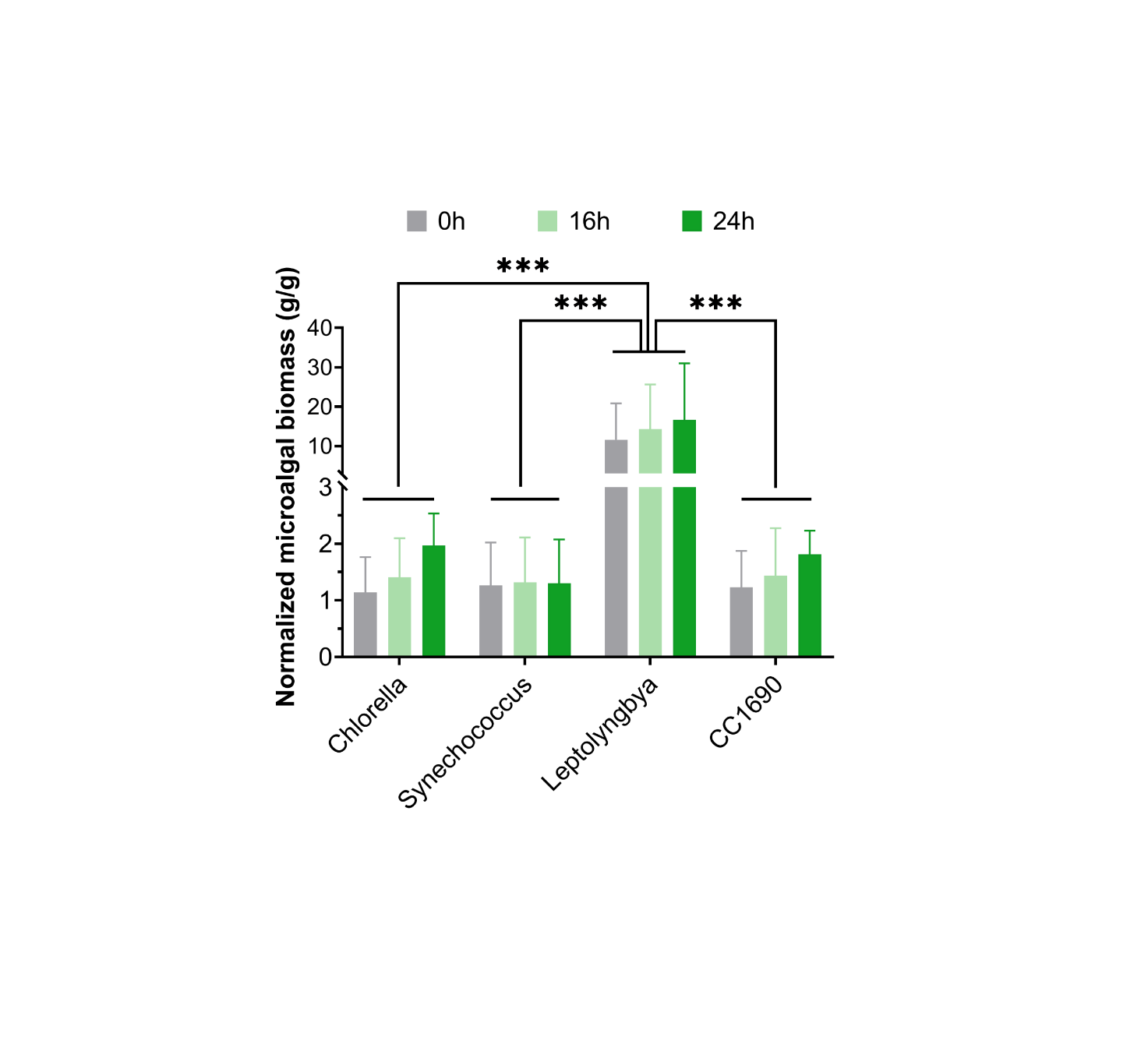


**Supplementary Figure 2. Normalized microalgal biomass for four algae species exposed to different light/dark regimens.** Data are expressed as mean ± SD. ***P ≤ 0.001.


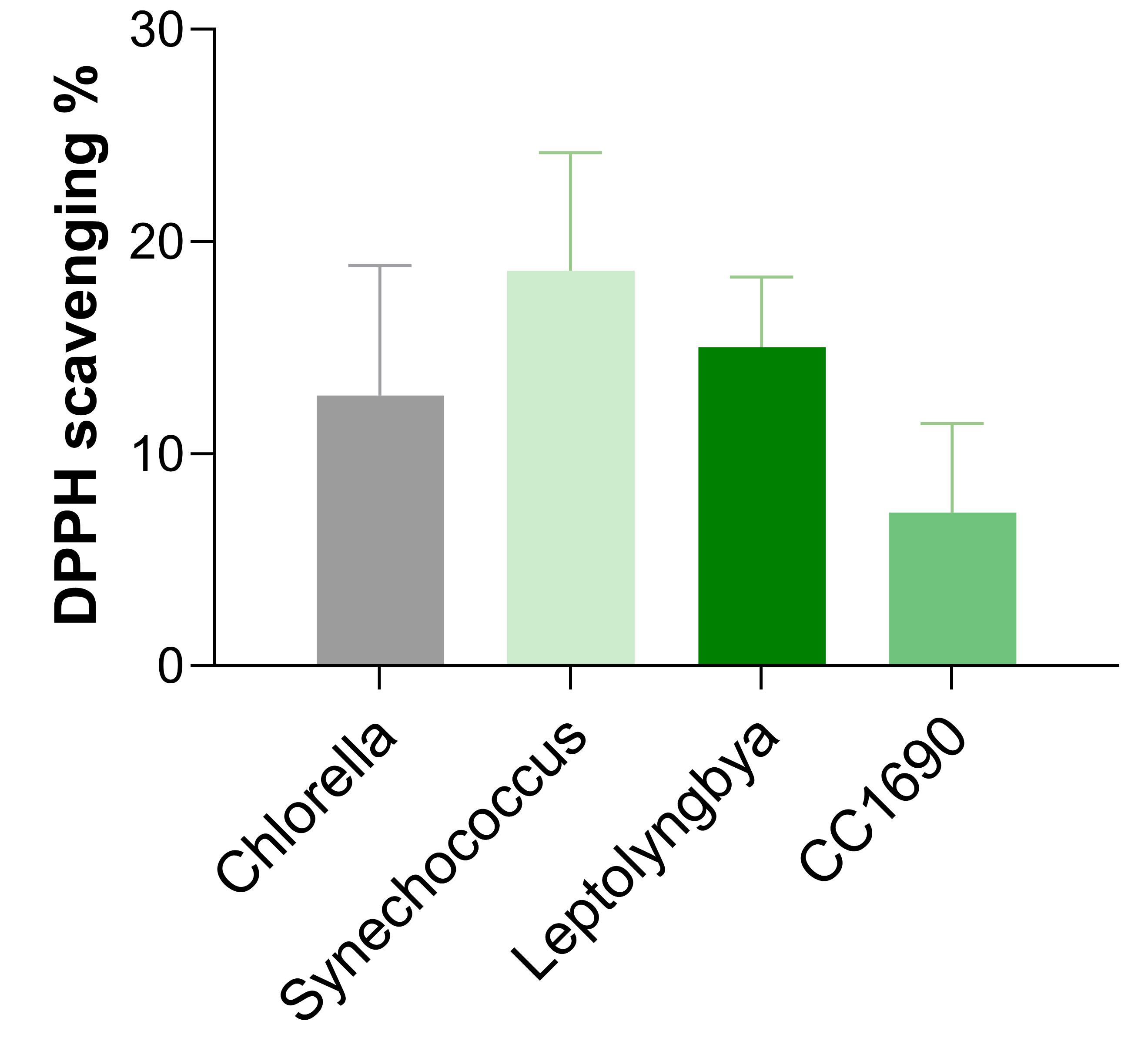


**Supplementary Figure 3. DPPH scavenging capacity of different microalgae EVs.** EVs obtained from standard 16h light cultivation protocol. Data expressed as mean ± SD.


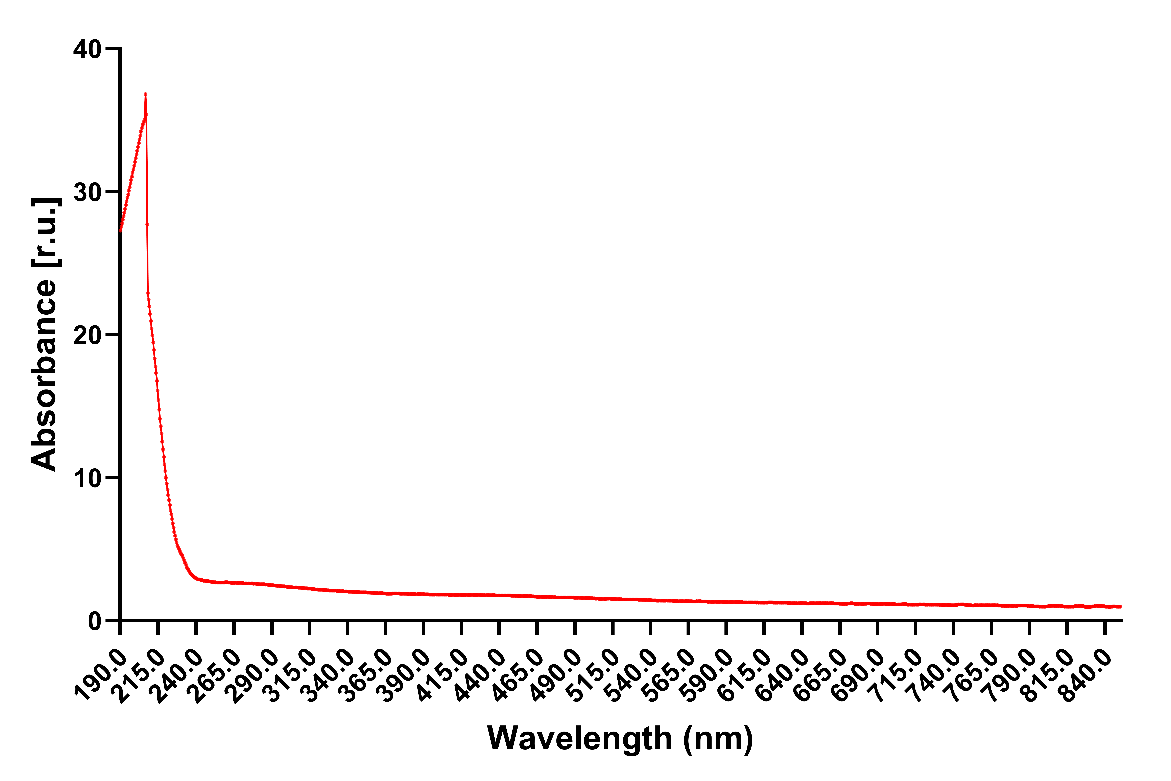


**Supplementary Figure 4. UV-Vis spectra of Lepto-EVs**


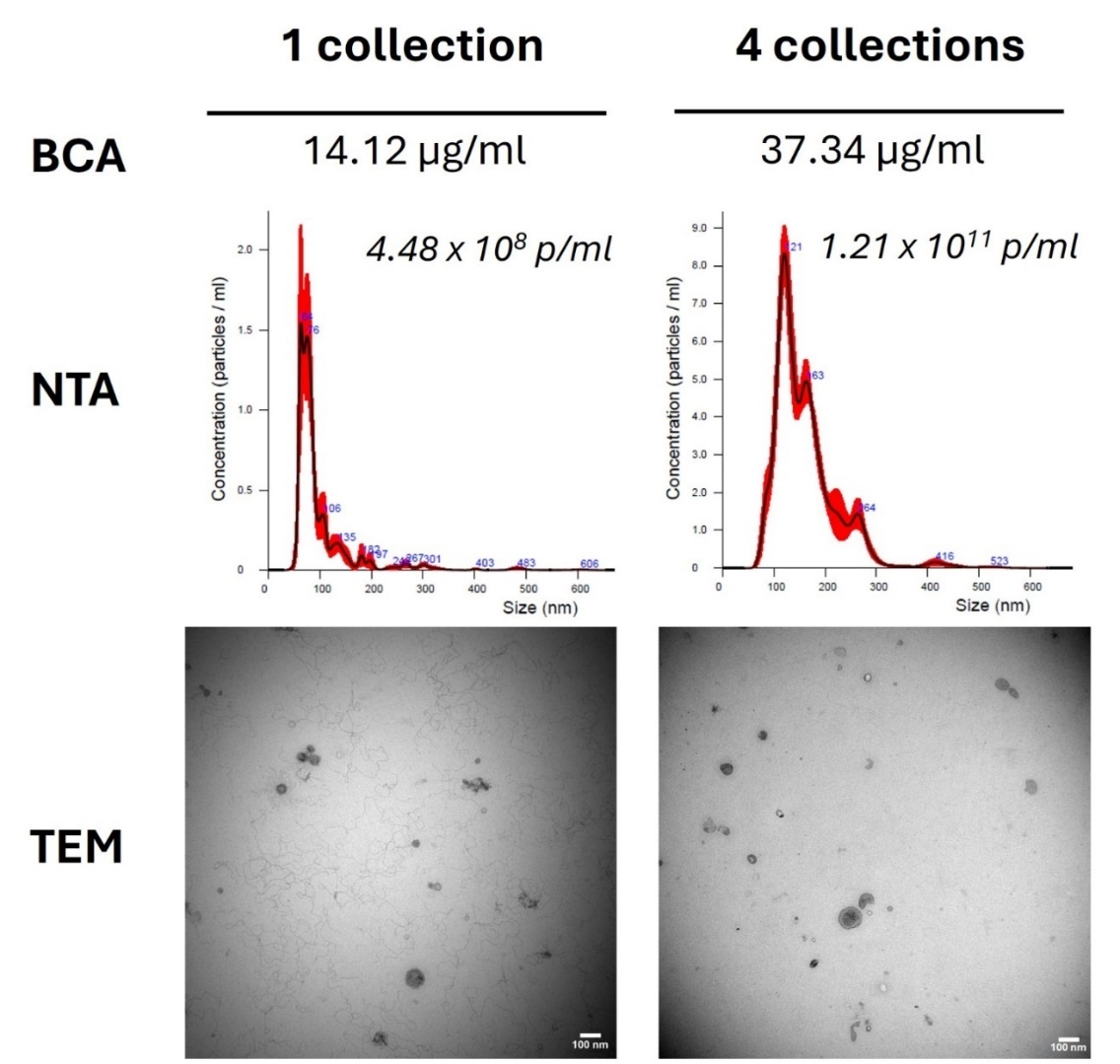


**Supplementary Figure 5. The effect of frequent *Leptolyngbya* medium collection on Lepto-EV yield.** EV protein content, nanoparticle concentration, and TEM.


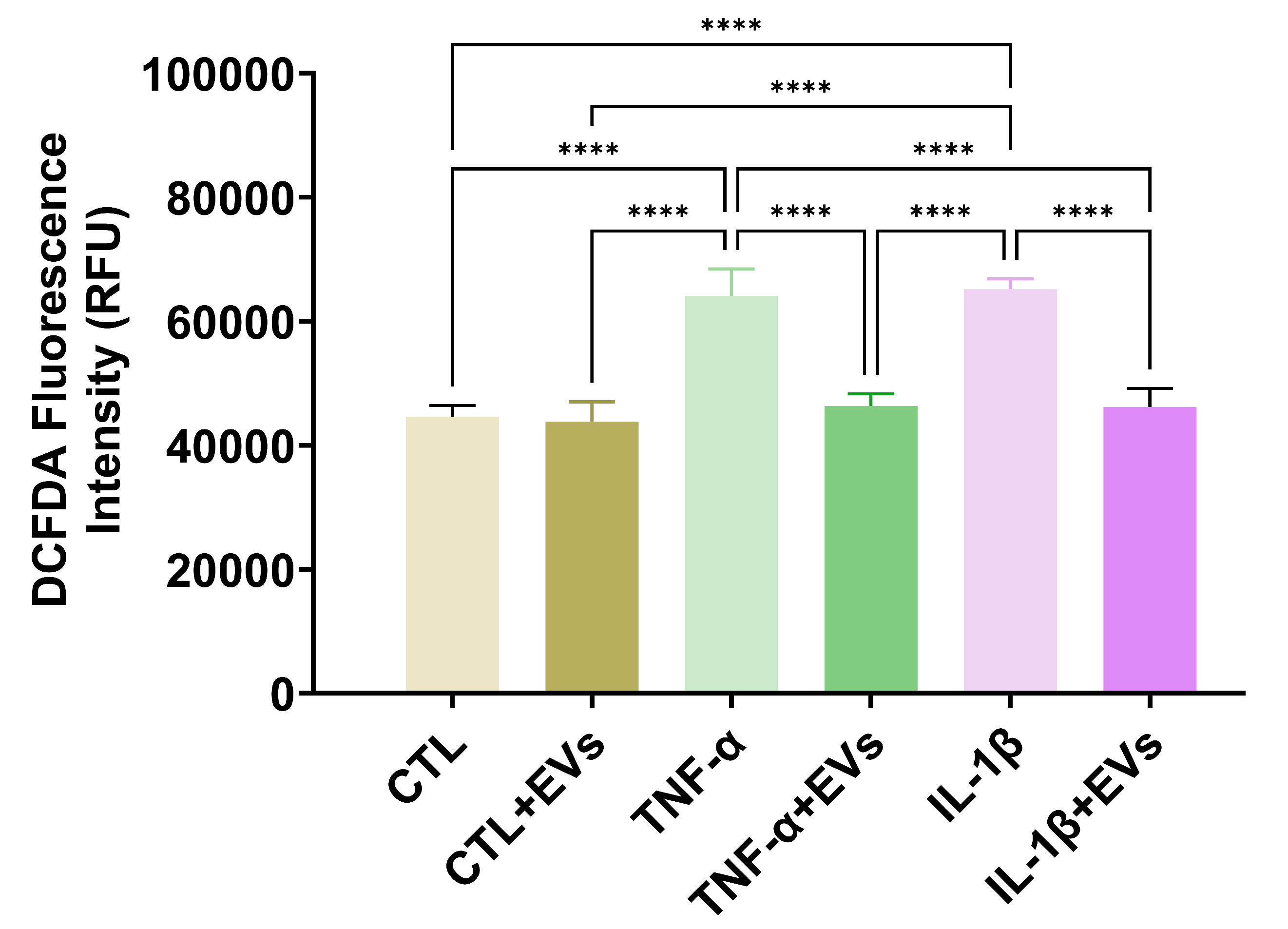


**Supplementary Figure 6. The fluorescence intensity of intracellular ROS levels in ATDC5 cells treated with DCFDA under untreated, TNF-α, IL-1β, TNF-α+EVs, IL-1β+EVs.** Data expressed as mean ± SD. ***P ≤ 0.001 and ****P ≤ 0.0001.
